# Information amount, accuracy, and relevance of generative artificial intelligence platforms’ answers regarding learning objectives of medical arthropodology evaluated in English and Korean queries in December 2023: a descriptive study

**DOI:** 10.3352/jeehp.2023.20.39

**Published:** 2023-12-28

**Authors:** Hyunju Lee, Soobin Park

**Affiliations:** College of Medicine, Hallym University, Chuncheon, Korea; Hallym University, Korea

**Keywords:** Artificial intelligence, Deep learning, Language, Self efficacy, Republic of Korea

## Abstract

**Purpose:**

This study assessed the performance of 6 generative artificial intelligence (AI) platforms on the learning objectives of medical arthropodology in a parasitology class in Korea. We examined the AI platforms’ performance by querying in Korean and English to determine their information amount, accuracy, and relevance in prompts in both languages.

**Methods:**

From December 15 to 17, 2023, 6 generative AI platforms—Bard, Bing, Claude, Clova X, GPT-4, and Wrtn—were tested on 7 medical arthropodology learning objectives in English and Korean. Clova X and Wrtn are platforms from Korean companies. Responses were evaluated using specific criteria for the English and Korean queries.

**Results:**

Bard had abundant information but was fourth in accuracy and relevance. GPT-4, with high information content, ranked first in accuracy and relevance. Clova X was 4th in amount but 2nd in accuracy and relevance. Bing provided less information, with moderate accuracy and relevance. Wrtn’s answers were short, with average accuracy and relevance. Claude AI had reasonable information, but lower accuracy and relevance. The responses in English were superior in all aspects. Clova X was notably optimized for Korean, leading in relevance.

**Conclusion:**

In a study of 6 generative AI platforms applied to medical arthropodology, GPT-4 excelled overall, while Clova X, a Korea-based AI product, achieved 100% relevance in Korean queries, the highest among its peers. Utilizing these AI platforms in classrooms improved the authors’ self-efficacy and interest in the subject, offering a positive experience of interacting with generative AI platforms to question and receive information.

## Graphical abstract

[Fig f4-jeehp-20-39]

## Introduction

### Background/rationale

After generative artificial intelligence (AI) appeared, including ChatGPT, on November 30, 2022, the use of generative AI for acquiring medical information has been reported frequently. In the first stage, the accuracy of answers of generative AI was the main topic, such as the ability to take a parasitology examination [1] or a national licensing examination [2]. Although generative AI platforms provide answers, they are only sometimes accurate or relevant. There are reports on the positive effects of using generative AI as a feedback tool in the classroom through active participation by medical students [3]; however, more accurate answers are needed to adopt generative AI platforms in medical schools’ classrooms. Furthermore, there are many generative AI platforms. It will be essential to test which platforms are better and to become familiar with the characteristics of each generative AI platform. There are local language-based generative AI platforms throughout the world. For example, Clova X, maintained by Naver Corp., has the advantage of allowing information retrieval in Korean. It is also meaningful to check whether local language-based AI systems are better because it is easier for medical students to enter prompts in their native language. These findings are anticipated to be instrumental for other medical students besides those at Hallym University, guiding the incorporation of generative AI platforms into the educational repertoire.

### Objectives

This study aimed to evaluate the performance of 6 generative AI platforms for medical arthropodology. We analyzed their performance by querying multiple generative AI platforms about specific learning objectives in medical arthropodology. A key focus was to ascertain the relative performance of these AI platforms when responding to inquiries in both Korean and English by measuring information amount, accuracy, and relevance. To achieve the objective of the study, we formulated 3 research questions as follows: first, which platform would provide the most information in both languages; second, which platform would be the best in its quality, including accuracy and relevance; and third, whether the answers of Korea-based generative AI platforms would be better when the prompt is in Korean?

## Methods

### Ethics statement

This was not a study of human populations. Therefore, neither approval by the institutional review board nor obtainment of informed consent was required.

### Study design

This was a descriptive study based on the authors’ interpretation of the generative AI platforms’ answers.

### Setting

A new approach was adopted in the parasitology course conducted from October 23 to December 1, 2023 at Hallym University. During this period, generative AI was integrated as a learning tool in the parasitology course. This course enrolled 72 students who interactively queried various generative AI platforms about predefined learning objectives in parasitology, receiving tailored responses. Subsequently, each student group critically evaluated the AI’s answers for information amount, accuracy, and relevance. Following students’ presentations, detailed feedback was provided by the course instructor, enhancing the student’s comprehension of the subject matter. This novel information-gathering and presentation method significantly increased student engagement in the parasitology course. Post-course, we endeavored to refine further and distill the information acquired from these sessions, focusing on assessing the responses of multiple generative AI platforms in medical arthropodology. From December 15 to 17, the 7 learning objectives of medical arthropodology were entered in queries to 6 generative AI platforms, including Bard (https://bard.google.com/chat) by Google Co., Bing (https://www.bing.com/) by Microsoft Co., Claude (https://claude.ai/) Claude by Entropic, Clova X (https://clova-x.naver.com/) by Naver Corp., GPT-4 (https://chat.openai.com/?model=gpt-4), and Wrtn (https://wrtn.ai/) by Wrtn Technologies, Inc. Clova X and Wrtn are products of Korean companies. Seven learning goals are presented in English and Korean. Below are learning objectives in English.

The students should be able to (1) define medical arthropodology; (2) explain the clinical importance of arthropods as vectors and pathogens that directly cause lesions; (3) explain the relationship between dust mites and allergies; (4) list the scientific name of the vector mites for Tsutsugamushi disease; (5) list the species of lice that live on humans; (6) explain how to treat lice infestation; and (7) explain the symptoms, diagnosis, and treatment of scabies.

### Variables

The outcome variables were information amount, accuracy, and relevance of answers by 6 generative AI platforms, including a comparison between prompts in 2 languages.

### Data source/measurement

We gathered answers from 6 different generative AI platforms by submitting 7 medical arthropodology class learning objectives. The input prompts were 7 learning objectives of the arthropodology class in English and Korean. Two authors read the answers by AI platforms and determined the score of information amount, accuracy, and relevance through discussion. A common test for information sources is a Currency, Relevance, Authority, Accuracy, and Purpose test [4]. Out of 5 criteria, only accuracy and relevance were included because we believed those 2 criteria were essential for students. Furthermore, the amount of information was added because it was thought to be critical for learning.

The quantity (i.e., the information amount) of each AI’s answers was counted.

We assessed each response’s accuracy and relevance by referencing academic sources, including parasitology textbooks in Korean, online textbooks in Medscape (https://www.medscape.com/), parasitology information at the United States Centers for Disease Control and Prevention (https://www.cdc.gov/), and journal articles in PubMed (https://pubmed.ncbi.nlm.nih.gov/) and KoreaMed (https://koreamed.org/). The accuracy of the answers was defined as the ratio of correct pieces of information to the total pieces of information provided. The relevance of each response to the query was the ratio of pertinent information to all information provided. The quality of answers from the 6 generative AI platforms was presented as accuracy and relevance. Generative AI platforms’ answer data and interpretations are available in Dataset 1, where irrelevant information is highlighted, and inaccurate information is indicated in red. In Dataset 2, the analysis data are presented.

### Bias

The generative AI platforms selected in this study were suggested in the guide booklet for the parasitology class. The topic for inquiry, medical arthropodology, was chosen because it was dealt with in the last hours of the parasitology class. Therefore, there was no bias in selecting topics.

### Study size

Sample size estimation was not required because all target materials were selected.

### Statistical methods

Descriptive statistics were used.

## Results

### Quantitative results of 6 generative AI platforms’ answers

**Fig. 1.**
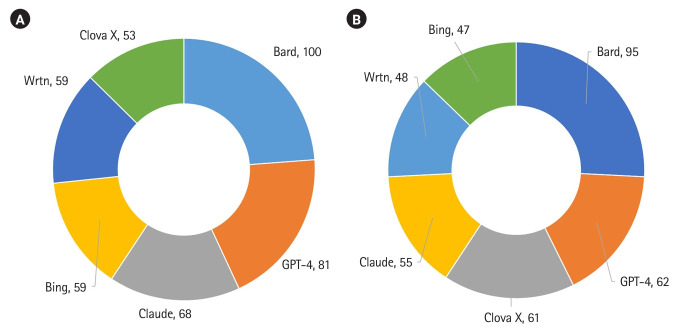
(A, B) Information amount, accuracy, and degree of relevance of 6 generative artificial intelligence platforms’ answers regarding the learning objectives of arthropodology regardless of language, as evaluated by 2 medical students in Korea, December 2023.

The information amount of 6 generative AI platforms was presented in Fig. 1 (Dataset 2). Bard consistently provided the most information, regardless of the language. The second-ranking was GPT-4 in both languages. Clova X, in Korean, was the 3rd.

### Quality of 6 AI platforms’ performance, including accuracy and relevance

#### Accuracy

**Fig. 2.**
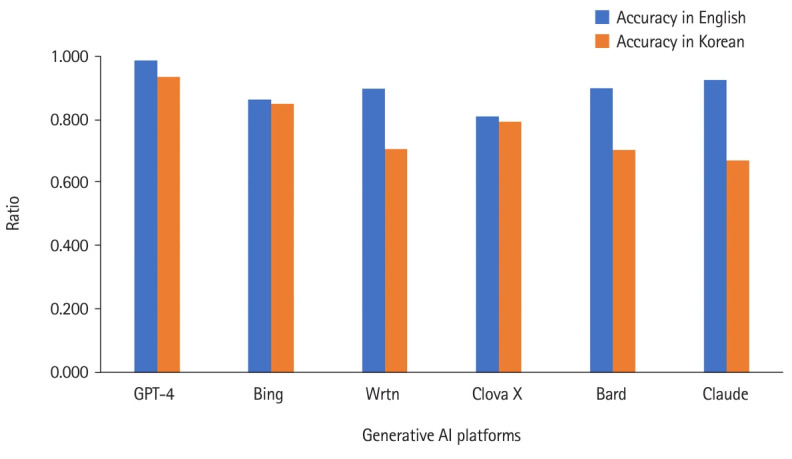
Accuracy of 6 generative artificial intelligence (AI) platforms’ answers regarding the 7 learning objectives of medical arthropodology in English and Korean queries, as assessed by 2 medical students in Korea, December 2023.

GPT-4 outperformed others in both languages. When queried in English, GPT-4 achieved nearly 99% accuracy. Its performance was also excellent for Korean queries. Bing was the 5th in English but 2nd in Korean. Clova X was the last in English queries but 3rd in Korean. Wrtn was the 4th in English and in Korean. Summing up the results of both languages, the ranking in descending order was as follows: GPT-4, Bing, Wrtn, Clova X, Bard, and Claude (Fig. 2, Dataset 2).

#### Relevance

**Fig. 3.**
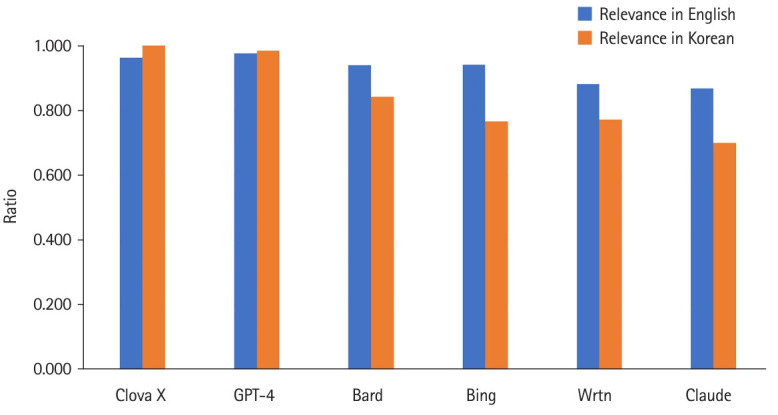
Relevance of 6 generative artificial intelligence (AI) platforms’ answers regarding the 7 learning objectives of medical arthropodology in English and Korean queries, as assessed by 2 medical students in Korea, December 2023.

Relevance varied with language. In English, GPT-4 was the most relevant, but in Korean, Clova X stood out. Summing up the results of both languages, the ranking in descending order was Clova X, GPT-4, Bard—Bing, Wrtn, and Claude (Fig. 3, Dataset 2).

### Comparison by language

We examined how the language used in AI queries affected the outcomes. When questions were asked in English, the responses were significantly better regarding information amount, accuracy, and degree of relevance. Therefore, it is evident that asking questions in English rather than Korean yields more optimal answers when using AI (Table 1).

### Performance of Korea-based generative AI platforms

Clova X and Wrtn presented relatively short information in English, but Clova X ranked 3rd when queried in Korean (Fig. 1). The accuracy of both AI platforms was 4th and 3rd when English and Korean queries were summed up (Fig. 2). The relevance of Clova X showed the best results in the Korean queries, with remarkably excellent results, and it was also 2nd in English; while Wrtn was the 4th ranking in Korean and 5th in English (Fig. 3)

## Discussion

### Key results

Google Bard consistently provided the most information in both languages when queried about learning objectives of medical arthropodology (Fig. 1). For accuracy, GPT-4 outperformed others in all languages (Fig. 1). In English queries, GPT-4 was the most relevant, but in Korean, Clova X stood out in its degree of relevance (Fig. 2). Prompts in English yielded more information, with higher accuracy and relevance, than Korean prompts (Table 1).

### Interpretation

#### Comparison of Korean and English answers of other AI platforms except for Clova

According to Figs. 2 and 3, it is evident that all AI platforms except Clova X delivered better responses in English than in Korean. GPT-4 demonstrated superior performance in responding to English queries. Interestingly, when posed questions in Korean, it exhibited a performance level almost equivalent to that in English. Moreover, compared with other AI platforms, its proficiency in Korean was remarkably high.

#### Clova X’s excellent performance to inquiries in Korean

Fig. 3 shows that Clova X was the only generative AI that performed best when asked in Korean rather than English. This performance confirms that Clova X has the advantage as a domestic Korean platform. It is confirmed that its understanding of Korean questions was high because it provided information with high relevance compared to other generative AI platforms. However, there could be higher accuracy and more information in Korean, which shows that Korean-based data needs improvement. Therefore, learning large amounts of accurate data is essential for developing a better Korea-based generative AI. In this study, Clova X also appeared to answer in Korean even though it was asked in English. In addition, it showed a weakness in English questions, such as answering that “it is not possible to answer” when asked in English.

#### Characteristics of each generative AI platform and their advantages

Bard: It provided the most information with a source link for each sentence, aiding in immediate accuracy checks. However, it needed help with understanding or accurately answering questions, particularly in Korean. Thus, users with basic knowledge can seek factual information using Bard.

Bing: It offered relatively little information. Like Bard, it included source links for easy truth verification.

Claude: It delivered decent responses in English, but the quality dropped significantly in Korean. It performed the worst among all AI platforms when asked in Korean.

Clova X: It performed better with Korean queries but needs improvement in accuracy and information amount. It is suitable for users seeking Korean-oriented responses.

GPT-4: It exhibited the best performance across all metrics, including language, information amount, accuracy, and relevance. Unique for being the only paid service in this study, it provided easy-to-read answers, distinguished by font size and boldness. It is an ideal platform for students or learners who need clear and concise information despite a slight shortage in quantity.

Wrtn: It had a relatively small amount of information and showed moderate performance in terms of accuracy and relevance, which can be competitive with other well-known generative AI platforms in Korean than English.

### Comparison with previous studies

Most articles on generative AI in medical education focused on the performance of generative AI, learning strategies including learning plans and learning materials, writing and research assistance, academic integrity concerns, accuracy and dependability, and potential detriments to learning [5]. For example, ChatGPT’s (GPT-3.5) knowledge and interpretation ability for parasitology examination were not yet comparable to those of medical students in Korea [1].

Cases of medical students’ use of generative AI in the classroom are not common in the literature. Park [3] introduced generative AI as a feedback tool in a school in Korea for a Leadership and Communication course. The potential effects of ChatGPT as a learning tool on 54 second-year pre-medical students in Korea were investigated, and the team project results showed no significant differences between the textbook group and the ChatGPT group [6]. Dental students asked questions to GPT-3.5, and its answers were compared to the answers by professors in a course on microbial pathogenesis in Sweden. In that study, GPT-3.5’s answers were relevant and correct (mean value=4.4/5.0) [7]. Although the suggestions of introducing generative AI platforms in medical education are abundant, example cases of using generative AI platforms in the classroom have yet to be published extensively.

There have been some comparative studies about the performance of multiple generative AI systems. Four large language models (Bard, New Bing, Claude, and ChatGPT4) were compared regarding urolithiasis. Claude consistently scored the highest. ChatGPT4 ranked second in accuracy, with a relatively stable output across multiple tests [8]. Torres-Zegarra et al. [2] evaluated the performance of 5 artificial intelligence chatbots, including Bard, Bing, Claude, GPT-3.5, and GPT-4, on the Peruvian National Medical Licensing Examination, where GPT-4 scored 86.7% and Bing scored 82.2%, followed by Bard and Claude. Bard, Bing AI, and ChatGPT-3.5 were compared for their performance regarding information on rhinoplasty [9]. Bard was the best, followed by ChatGPT-3.5 and Bing. These studies show that the performance of generative AI platforms varies depending on the system and the topics of inquiry.

### Limitations

The answer of each generative AI platform may vary according to the prompt content and time of input of the prompt due to the growing speed of each AI. The present results cannot be guaranteed in the future. The reproducibility of study results with generative AI is the main limitation. Therefore, answer data are provided in Dataset 1. The interpretation of accuracy was done by 2 authors who were medical students. Although they passed the parasitology course and referred to many references, their decisions may not always be correct. The characteristics of each generative AI platform and their advantages are only applicable to the present study. The findings cannot be extrapolated or generalized in other topics.

### Suggestion for further studies

Medical arthropodology is a highly specialized field. Therefore, more general conclusions could be generated by analyzing more common medical subjects, such as heart diseases, endocrine diseases, or coronavirus disease 2019, and by inputting a larger number of learning objectives. Furthermore, pre-training with big data in a specialized field, such as parasitology, would make it possible to generate more accurate answers.

### Implications

#### Is assessing the accuracy and relevance of AI’s answers beneficial for learning?

Through this new class format in parasitology, we actively engaged in roles as independent learners. In medical education, there may often be a discrepancy between the publication year of textbooks and when the class is held, resulting in a gap in the methods and theories currently applied in clinical practice. We gained more practical and applicable knowledge by checking the accuracy of the latest information provided by the generative AI against recent research articles, books, and academic materials. This also enhanced self-efficacy, increasing interest in the subject matter.

#### How will we utilize generative AI in the future?

As future physicians, continuous learning to amass diverse knowledge and skills, including AI, is essential to stay calm in the face of technological advances and stay caught up. Particularly, if past medical practices were marked by information asymmetry, the advent of AI will help reduce this gap by offering a variety of information to patients. In such scenarios, patients can present and inquire about knowledge acquired through AI while visiting physicians. Integrating AI with professional expertise will be crucial for providing patients with more accurate and precise information.

When comparing the learning capabilities of humans and AI, AI undoubtedly excels in the speed of processing a vast amount of information, as presented in the present study. However, the information provided by AI still needs to be more accurate. Nonetheless, generative AI cannot effectively judge the reliability and accuracy of information sources. As physicians or medical experts, our advantage over generative AI lies in the ability to discern between accurate and inaccurate information based on professional knowledge. This skill of discrimination is a crucial competency that physicians and medical scientists must possess, and it will be one of the key reasons why generative AI may not replace us.

### Conclusion

When comparing the performance of 6 generative AI platforms regarding the learning objectives of medical arthropodology, GPT-4 outperformed the others. A Korea-based company’s generative AI, Clova X, showed 100% relevance to the queries in Korea, which is the best performance out of the 6 generative AI platforms. The experience of using generative AI in the classroom enhanced the authors’ self-efficacy, which led to a heightened interest in the subject matter.

## Figures and Tables

**Figure f4-jeehp-20-39:**
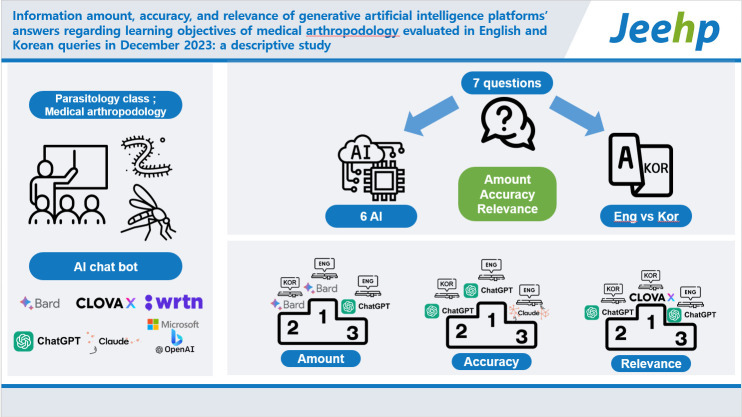


**Table 1.** Comparison of information amount, accuracy, and degree of relevance of 6 generative artificial intelligence platforms’ answers regarding the learning objectives of medical arthropodology according to language assessed by 2 medical students in Korea, December 2023

	English	Korean
Information amount	418	377
Accuracy	0.904	0.771
Degree of relevance	0.929	0.853

## Footnotes

**Data availability**
Data files are available from Harvard Dataverse: https://doi.org/10.7910/DVN/7M0R9Z Dataset 1. Answers of 6 generative artificial intelligence platforms to the learning objectives of medical arthropodology during the parasitology class according to queries in English and Korean and interpretation by 2 medical students in Korea from December 15 to 18, 2023.

Dataset 2. Analysis data derived from Dataset 1, with answers of 6 generative artificial intelligence platforms to the learning objectives of medical arthropodology during the parasitology class according to queries in English and Korean and interpretation by 2 medical students in Korea from December 15 to 18, 2023.

## Supplementary materials

Supplement 1. Audio recording of the abstract.

## References

[1] Huh S (2023). Are ChatGPT’s knowledge and interpretation ability comparable to those of medical students in Korea for taking a parasitology examination?: a descriptive study. J Educ Eval Health Prof.

[2] Torres-Zegarra BC, Rios-Garcia W, Nana-Cordova AM, Arteaga-Cisneros KF, Chalco XC, Ordonez MA, Rios CJ, Godoy CA, Quezada KL, Gutierrez-Arratia JD, Flores-Cohaila JA (2023). Performance of ChatGPT, Bard, Claude, and Bing on the Peruvian National Licensing Medical Examination: a cross-sectional study. J Educ Eval Health Prof.

[3] Park J (2023). Medical students’ patterns of using ChatGPT as a feedback tool and perceptions of ChatGPT in a Leadership and Communication course in Korea: a cross-sectional study. J Educ Eval Health Prof.

[4] Blakeslee S https://commons.emich.edu/loexquarterly/vol31/iss3/4.

[5] Preiksaitis C, Rose C (2023). Opportunities, Challenges, and Future Directions of Generative Artificial Intelligence in Medical Education: Scoping Review. JMIR Med Educ.

[6] Hwang JY (2023). Potential effects of ChatGPT as a learning tool through students’ experiences. Med Teach.

[7] Hultgren C, Lindkvist A, Ozenci V, Curbo S (2023). ChatGPT (GPT-3.5) as an assistant tool in microbial pathogenesis studies in Sweden: a cross-sectional comparative study. J Educ Eval Health Prof.

[8] Song H, Xia Y, Luo Z, Liu H, Song Y, Zeng X, Li T, Zhong G, Li J, Chen M, Zhang G, Xiao B (2023). Evaluating the performance of different large language models on health consultation and patient education in urolithiasis. J Med Syst.

[9] Seth I, Lim B, Xie Y, Cevik J, Rozen WM, Ross RJ, Lee M (2023). Comparing the efficacy of large language models ChatGPT, BARD, and Bing AI in providing information on rhinoplasty: an observational study. Aesthet Surg J Open Forum.

