# Supplementary material for: Information amount, accuracy, and relevance of generative artificial intelligence platforms’ answers regarding learning objectives of medical arthropodology evaluated in English and Korean queries in December 2023: a descriptive study
Source: J Educ Eval Health Prof. 2023 Dec 28;20:39. doi: 10.3352/jeehp.2023.20.39 (PMC11893187; doi:10.3352/jeehp.2023.20.39)
Supplement: Supplementary file 1 [file jeehp-20-39-dataset1.docx]

Dataset 1. Answers of 6 chatbots to 7 learning goals of arthropology in English and Korean with an interpretation by the authors

탐구내용

1. 의용절지동물학을 정의 내릴 수 있어야한다.

<한글 - GPT 4.0>

"의용절지동물학"은 의료 분야에서 절지동물을 활용하는 학문을 말합니다. 이 분야는 주로 절지동물의 생물학적 특성이 인간의 건강과 의학에 어떻게 적용될 수 있는지를 연구합니다. / 절지동물에는 곤충, 거미류, 갑각류 등이 포함되며, 이들은 다양한 의학적, 생물학적 연구에 중요한 역할을 합니다. /

의용절지동물학의 주요 분야는 다음과 같습니다:

- **질병 연구와 치료**: 일부 절지동물은 인간 질병의 모델로 사용되며, 이들을 통해 질병의 발달과정, 유전학, 그리고 잠재적 치료법을 연구합니다. 예를 들어, 과일파리는 유전학 연구에 널리 쓰이며, 일부 해충은 전염병의 전달자로 연구됩니다. /
- **약품 개발**: 절지동물은 새로운 약물이나 치료법 개발에 기여합니다. 예를 들어, 뱀독에서 유래한 약품은 혈액 응고 치료에 사용되며, 거미독은 통증 완화 약물 개발에 활용됩니다. /
- **생물 의학 재료**: 절지동물에서 추출한 물질은 의학적 물질로 개발될 수 있습니다. 예를 들어, 게의 껍질에서 추출한 키틴은 상처 치료제나 수술용 봉합사에 사용됩니다. /
- **환경 건강과 공중 위생**: 일부 절지동물은 환경의 건강 상태를 모니터링하는 데 사용됩니다. 또한 해충 관리를 통해 질병을 예방하는 공중 위생의 한 부분으로도 중요합니다. /

이 분야는 인간의 건강과 직결되어 있으며, 지속적인 연구를 통해 새로운 치료법과 약품 개발에 기여하고 있습니다.

<영어 - GPT 4.0>

Medical Arthropodology is a specialized field of study that focuses on arthropods (a group of invertebrate animals that includes insects, arachnids, crustaceans, and others) and their relationship to human health and disease. / This field is an intersection of entomology (the study of insects) and medicine, and it has significant implications for public health, epidemiology, and the development of new medical treatments. / Key aspects of Medical Arthropodology include:

- **Disease Transmission**: One of the primary focuses is on arthropods that serve as vectors for disease. This includes studying how certain mosquitoes transmit malaria, dengue fever, Zika virus, and other diseases. Tick-borne illnesses like Lyme disease are also a significant area of study. /
- **Venom Research**: The study of venom from arachnids (like spiders and scorpions) and other arthropods for medical applications, such as the development of antivenoms or potential therapeutic uses of venom components in pain management, cancer treatment, and other areas. /
- **Allergy and Hypersensitivity**: Investigating allergic reactions caused by arthropods, such as bee stings or dust mite allergies, and developing treatments or preventative strategies. /
- **Parasitology**: Some arthropods are parasites or hosts to parasitic diseases affecting humans, and their study is crucial in understanding and controlling these health issues. /
- **Environmental Health**: Arthropods play a role in the health of our environment, which in turn affects human health. Studying their roles in ecosystems can help in understanding and managing issues like pest control in a way that minimizes harm to both humans and the environment. /

Medical Arthropodology is vital in the context of global health, particularly in managing and preventing diseases that can have widespread and significant impacts on human populations. Researchers in this field work closely with public health officials, epidemiologists, and medical professionals to monitor, control, and develop treatments for diseases associated with arthropods. /

<한글 - Claude Ai>

의용절지동물학은 절지동물을 이용하여 의학 및 생물학 연구를 하는 학문입니다./ 주요 내용은 다음과 같습니다:

절지동물(주로 원숭이, 개, 고양이 등)을 이용하여 인체 생리학, 약리학, 질병 연구 등을 수행 /

절지동물을 모델로 하여 인체 질환의 발생 기전 및 치료법 개발/

신약 및 의료기기의 안정성과 유효성 검증을 위한 동물실험 수행/

절지동물의 해부학, 생리학, 유전학 등 기초 의학 지식 습득/

의용절지동물학은 동물복지와 윤리적 쟁점들이 수반되므로, 연구 수행 시 동물실험 윤리기준을 준수하는 것이 중요합니다. 최근에는 동물실험 대체 방법도 주목받고 있습니다./

⇒ 절지동물의 종류로 원숭이, 개, 고양이를 제시하여 틀린 정보를 포함한다. 또 의용절지동물학 연구 수행 시 준수해야 할 윤리기준은 의용절지동물학의 정의와는 관련성이 떨어지는 정보이다.

또 인체 질환의 발생 기전 및 치료법 개발에 절지동물이 모델이 되고, 신약 및 의료기기의 안정성과 유효성 검증을 위한 동물실험에 수행된다는 내용은 “임상 기생충학(서울의대 출판)”을 확인하였을 때 전혀 찾아볼 수 없었고 동물실험 시 절지동물을 피실험개체로 이용하는 자료도 찾아볼 수 없어 두 내용이 부정확하고 의용절지동물학과 관련이 없는 내용이라고 판단하였다.

<영어 - Claude Ai>

Medical arthropodology refers to the study of arthropods for medical and biological research purposes. / The key aspects include:

Using arthropods (mainly insects, arachnids, crustaceans) as model organisms to study human physiology, pharmacology, diseases, etc./

Investigating the mechanisms of human diseases and developing treatments using arthropod models. /

Conducting animal testing on arthropods to evaluate the safety and efficacy of new drugs and medical devices. /

Learning the fundamental anatomy, physiology, genetics of arthropods to acquire basic medical knowledge. /

Medical arthropodology involves ethical concerns and animal welfare issues. It is important to follow ethical guidelines for animal testing when conducting research. Recently, alternative methods to replace animal testing are also gaining attention.

⇒ 한글버전 과는 달리 영어버전에서는 절지동물이 원숭이, 개, 고양이를 포함한다는 오류를 범하지 않고 정확한 정보를 제공하였다. 기본적으로 제공하는 정보는 한글 버전의 답변과 같은 구조였으나 정보의 세부사항의 정확도가 차이가 나는 것으로 보인다.

한글버전의 답변과 같이 의용절지동물의 신약개발과 인간의학 연구 시 모델로 사용되는 것은 의용절지동물학과 관련성이 적고 부정확한 정보로 판단하였다.

<한글 - Clova X>

의용절지동물학은 절지동물과 절지동물에 의한 질병을 연구하는 학문으로, 다음과 같은 내용을 포함합니다. /

1. 절지동물의 분류와 생태 /

2. 절지동물의 질병과 감염 /

3. 절지동물의 예방과 치료 /

의용절지동물학은 의용곤충학과 밀접한 관련이 있으며, 절지동물의 종류와 특성에 따라 다양한 분야에서 연구가 이루어지고 있습니다. 예를 들어, 곤충류는 농업, 의학, 환경 등 다양한 분야에서 연구가 이루어지고 있으며, 게, 새우류는 식품 산업에서 중요한 역할을 합니다. /

의용절지동물학은 인간의 건강과 생태계에 미치는 영향을 파악하고, 이를 예방하고 치료하는 데 중요한 역할을 합니다. / 또한, 절지동물의 생태와 진화를 연구하여 생물학적 다양성을 이해하는 데에도 큰 도움을 줍니다. /

⇒절지동물의 예방과 치료가 아니라 절지동물로 인해 발생하는 질병과 치료라고 표현하는 것이 더 정확하다고 판단하여 “절지동물의 예방과 치료”를 부정확한 정보로 판단하였다.

<영어 - Clova X>

한글로 질문하였을 때와 동일한 답변이 제공되었다.

<한글 - Bing Ai>

의용절지동물학은 인체에 영향을 미치는 절지동물과 그들이 매개하는 감염병에 대해 연구하는 학문입니다. / 절지동물은 다리가 관절로 구분되어 있는 동물로, 곤충, 거미, 진드기, 벼룩, 벌, 개미 등이 있습니다. / 의용절지동물학은 절지동물의 분류, 생태, 독성, 유전, 알레르겐, 감염병 등에 관한 지식을 제공하고, 인체의 건강을 보호하고 질병을 예방하고 치료하는 방법을 연구합니다. / 의용절지동물학은 의학, 수의학, 생물학, 환경학 등과 밀접한 관련이 있습니다. /

⇒ “임상 기생충학(서울의대 저)”를 보면 절지동물은 다리 외의 여러 부속지들이 모두 마디라고 기재되어 있다. 다리가 관절로 구분되어있는 동물이라는 표현은 Arthropod 원어의 뜻인 것은 맞긴 하다. 하지만 사람도 다리가 관절로 구분되어 있으나 절지동물로 분류되지 않는 것처럼, 다리가 관절로 구분되어 있는 것은 절지동물의 필요조건이기는 하나 충분조건이 될 수 없기 때문에 완전히 틀린 말이라고 하긴 어렵지만 이용자로 하여금 오해를 할 수 있다는 점에서 정확성이 떨어지는 정보로 판단했다.

<영어 - Bing Ai>

Medical arthropodology is a branch of arthropodology that studies the impact of arthropods on human health and disease./ Arthropods are animals with jointed legs, such as insects, spiders, ticks, fleas, bees, ants, etc./ Medical arthropodology investigates the classification, ecology, toxicity, genetics, allergens, infectious diseases, and other aspects of arthropods that affect human health and well-being./ It also develops methods to protect, prevent, and treat arthropod-borne diseases and infestations. / Medical arthropodology is closely related to medicine, veterinary medicine, biology, environmental science, and other disciplines.

⇒한글 답변과 거의 같은 내용을 제공하고 있다. 한글버전과 마찬가지로 Arthropods의 정의에 대해 정확성이 떨어지는 정보를 제공한다고 판단하였다.

<한글 - Bard>

의용절지동물학(medical entomology)은 절지동물(곤충, 거미, 갑각류 등)이 인간에게 미치는 영향을 연구하는 학문입니다. / 절지동물은 질병을 매개하거나, 직접적인 피해를 입히거나, 알레르기를 유발하는 등 다양한 방법으로 인간의 건강에 영향을 미치기 때문에, 의용절지동물학은 질병의 예방과 치료, 그리고 건강 증진에 중요한 역할을 합니다. /

의용절지동물학의 주요 연구 분야는 다음과 같습니다.

질병 매개 : 절지동물은 말라리아, 뎅기열, 리슈마니아증, 황열병 등 다양한 질병을 매개합니다. 의용절지동물학은 이러한 질병의 매개체인 절지동물을 연구하여 질병의 예방과 치료에 기여합니다. /

직접적인 피해 : 절지동물은 사람의 피를 빨아먹거나, 피부에 상처를 입히거나, 알레르기를 유발하는 등 직접적인 피해를 입힐 수 있습니다. 의용절지동물학은 이러한 피해를 예방하고 치료하는 방법을 연구합니다. /

의약품 개발 : 절지동물은 항생제, 항암제, 진통제 등 다양한 의약품의 원료가 됩니다. 의용절지동물학은 절지동물에서 유래한 의약품을 개발하는 연구를 수행합니다. /

의용절지동물학은 의학, 생물학, 환경학 등 다양한 분야와 연계되어 발전하고 있습니다. 최근에는 기후 변화와 환경 오염으로 인해 절지동물의 분포와 행동이 변화하고 있어, 의용절지동물학의 중요성이 더욱 커지고 있습니다. /

⇒의용절지동물학을 medical entomology, 즉 의용 곤충학이라는 뜻의 영어 단어로 설명하여 부정확한 것으로 판단함. 의약품 원료로서 절지동물을 이용한다는 문장의 정확성이 의심되어 조사해본 결과, (Al-Ani I, Zimmerman S., Reichling J, Wink M (2015) Pharmacological synergism of bee venom and melittin with antibiotics and plant secondary metabolites against multi-drug resistant microbial pathogens. Phytomedicine 22: 245-255.

Ramos-Elorduy de Concini J, Pino Moreno JM (1988) The utilization of insects in the empirical medicine of ancient Mexicans. Journal of Ethnobiology 8: 195–202.

El-Wahab SDA, Eita WLH (2015) The effectiveness of live bee sting acupuncture on depression. Journal of Nursing and Health Science 4: 19-27.

Falco A, Barrajon-Catalan E., Menendez-Gutierrez MP, Coll J, Mocol V, Estepa A (2013) Melittin-loaded immunoliposomes against viral surface proteins, a new approach to antiviral therapy. Antiviral Research 97: 218-221.

Sarfo-Poku C, Eshun O, Lee KH (2016) Medical application of scorpion venom to breast cancer: A mini-review. Toxocon 122: 109-112.) 를 reference로 사용한 article(<https://www.terminix.com/blog/science-nature/arhropod-venom-use-in-medicine/>)에서 절지동물의 독을 의약품으로 사용하는 연구가 진행되고 있음을 알 수 있었다. 하지만 절지동물 그 자체를 의약품으로 이용하는 경우는 찾아볼 수 없었고, 해당 ai의 답변은 이용자로 하여금 혼란을 줄 수 있는 정확도가 떨어지는 답변이라고 판단하였다. 그리고 관련성에 관하여도, 절지동물에서 유래한 약품을 연구하는 학문이 의용절지동물학에 포함되는 것을 “임상 기생충학(서울의대 저)”에서는 찾아볼 수 없었으므로 관련성도 떨어지는 정보로 판단하였다.

<영어 - Bard>

Medical arthropodology is the study of the interaction between arthropods and humans, with a focus on the role of arthropods in disease transmission and control./ Arthropods are a large and diverse group of animals that includes insects, spiders, ticks, and mites./ Many arthropods are vectors of diseases, meaning that they can transmit pathogens from one infected host to another./ Medical arthropologists study the biology, ecology, and behavior of arthropods in order to develop and implement strategies for preventing and controlling vector-borne diseases./

Medical arthropodologists also study the impact of arthropods on human health in other ways. For example, they study arthropod-borne allergies, envenomations, and parasitic infections./ They also study the role of arthropods in forensic science and public health./

Here are some examples of topics that medical arthropologists study:

The biology, ecology, and behavior of arthropod vectors of diseases such as malaria, dengue fever, yellow fever, and Zika virus

The development and implementation of vector control measures, such as insecticide spraying and mosquito bed nets. /

⇒한글과 달리 의용절지동물학의 영어 term을 올바르게 기재하였다. 이 점에서 영어로 질문하는 것이 한글로 질문하는 것보다 나음을 알 수 있었다. 또 한글로 질문하였을 때 답변에 있었던 “절지동물의 의약품으로서의 이용”은 정확성과 관련성이 떨어진다고 판단하였는데, 영어로 질문하였을 때는 이를 언급하지 않았다.

<한글 - Wrnt>

의용절지동물학은 의학적 목적으로 사람을 돕기 위해 훈련된 동물들의 사용과 관련된 학문입니다./ 이 학문은 주로 사람들의 신체적, 정신적, 사회적인 필요에 부합하는 방식으로 동물들을 훈련시키고 활용하는 방법에 대해 연구합니다./ 의용절지동물학은 주로 장애인 보조견, 재활치료를 위한 말과 수영 치료, 정신건강 치료를 위한 반려동물 등 다양한 분야에서 활용됩니다. 이를 통해 동물들은 사람들의 삶의 질을 향상시키고 도움을 주는 중요한 역할을 수행합니다. /

⇒ 의용절지동물학을 “훈련된 동물 사용과 관련된 학문”으로 잘못 해석하여 완전히 부정확하고 관련성 없는 답변을 내놓았다.

<영어 - Wrnt>

Medical arthropodology is a branch of science that focuses on the study of arthropods (insects, ticks, mites, etc.) that are of medical importance. / It encompasses the understanding of arthropod-borne diseases, their transmission, and the interactions between arthropods and human health./ Medical arthropodology plays a crucial role in the field of public health as it helps in the prevention, control, and management of vector-borne diseases./ Researchers in this field study the life cycles, behavior, ecology, and control methods of medically significant arthropods to develop effective strategies for disease prevention and vector control./ This knowledge is essential for understanding the spread of diseases such as malaria, dengue fever, Lyme disease, and many others that are transmitted by arthropods. /

⇒한글은 완전히 틀린 정보를 답변했던 반면 영어로 질문하였을 때는 정확하고 관련성이 높은 답변을 제시하였다.

2. 의용절지동물의 임상 중요성을 매개체로서와 직접 매개체로서와 직접병변을 일으키는 기생체로서 설명할 수 있어야한다.

<한글 - GPT 4.0>

- 의용절지동물의 임상 중요성은 주로 두 가지 방식으로 나타납니다: 매개체로서의 역할과 직접 병변을 일으키는 기생체로서의 역할입니다.
- 1. **매개체로서의 역할**: 이 경우, 절지동물은 병원체를 사람에게 전달하는 매개체로 작용합니다. 이들은 다음과 같은 방식으로 질병을 전파할 수 있습니다:/
- - **모기**: 말라리아, 뎅기열, 지카 바이러스 등의 전파에 책임이 있습니다. 이들은 병원체를 품은 상태로 사람을 물어 감염을 일으킵니다./
- - **진드기**: 라임병, 쯔쯔가무시병과 같은 질병을 전파합니다. 감염된 숙주의 혈액을 흡수한 후, 다른 숙주에게 병원체를 전달할 수 있습니다/.
- 2. **직접 병변을 일으키는 기생체로서의 역할**: 이 경우, 절지동물 자체가 인간에게 직접적인 건강 문제를 일으킵니다. /예를 들면:
- - **먼지진드기**: 이들은 집먼지 알레르기의 주요 원인으로, 호흡기 알레르기나 아토피 피부염을 유발할 수 있습니다./
- - **이와 진드기**: 이들은 피부에 기생하면서 가려움, 발진 등의 피부 문제를 일으키는 원인이 됩니다./
- 이러한 방식으로 의용절지동물은 직접적으로나 간접적으로 인간의 건강에 큰 영향을 미칩니다. 이들의 역할을 이해하고 적절히 관리하는 것은 공중보건과 질병 예방에 있어 중요합니다.

⇒매개체로서의 역할을 틀리게 제시한 바는 없으나, 임상기생충학(서울의대 저)에 나와있는 매개체로서의 역할에 대한 기계적 전파와 생물학적 전파의 분류가 나와있지 않은 것이 아쉽게 느껴진다. 매개체로 작용하는 절지동물이 모기와 진드기만 있는 것이 아님에도, “예를 들어”나 “등”과 같은 표현 없이 두 절지동물만 제시한 것은 이용자로 하여금 잘못된 정보를 맞다고 생각할만 하다고 판단하여 정확도가 부족한 답변이 0.5개 있는 것으로 산정하기로 하였다.

<영어 - GPT 4.0>

In the context of medical and clinical importance, arthropods can be understood in two distinct roles: as a medium (or vector) for disease transmission and as parasites that directly cause lesions./ Here's an explanation of each role:

1. **Arthropods as a Medium for Disease Transmission**: In this role, arthropods serve as vectors, transmitting diseases from one host to another. This aspect is vital in understanding the spread of many infectious diseases./

- **Disease Vectors**: Many arthropods, notably mosquitoes and ticks, are known for their role in transmitting pathogens./ For example, mosquitoes are vectors for malaria, dengue fever, Zika virus, and yellow fever. Ticks can transmit Lyme disease and Rocky Mountain spotted fever. These diseases are of significant clinical importance because they can lead to severe illness and even death in humans./

- **Mechanism of Transmission**: The transmission usually occurs when the arthropod feeds on human blood. The pathogens (like viruses, bacteria, or parasites) residing in the arthropod are transferred to humans during this process./

2. **Arthropods as Parasites Causing Direct Lesions**: In this role, arthropods directly affect human health by causing physical harm or lesions through their parasitic activities. /

- **Direct Skin Infestations**: Certain arthropods, such as scabies mites (Sarcoptes scabiei) or lice, infest human skin or hair. Scabies mites burrow into the skin to lay eggs, leading to intense itching and a characteristic rash. Lice can infest scalp hair, causing itching and irritation. /

- **Allergic Reactions and Secondary Infections**: The physical presence of these arthropods and their activities on the skin can lead to allergic reactions. Scratching due to itching can also lead to secondary bacterial infections, which can complicate the clinical picture. /

In both cases, the clinical importance of these arthropods lies in their ability to affect human health significantly./ For vectors, controlling their population and preventing bites are crucial in disease prevention. /For direct parasitic infestations, diagnosis and appropriate treatment to eradicate the parasites are key./ Public health measures, awareness, and research into controlling these arthropods are essential parts of managing the health risks they pose./

⇒ 영문 GPT 4.0은 나의 질문 형식에 맞추어 절지동물을 분류해준 것이 인상적이었다. 한글 답변에서 있었던 아쉬움과 달리 Disease vector로서 모기와 진드기가 “notably known for their role as a pathogen”이라는 표현으로 이외에도 많은 vector가 있음을 시사해주었다는 것이 한글 답변보다 나은 부분이었다. 또 기생체로서의 절지동물에 대하여도 정확한 정보만을 제공하였다.

<한글 - Claude Ai>

의용절지동물의 임상적 중요성을 설명하자면 다음과 같습니다.

첫째, 매개체로서의 중요성입니다. 모기, 진드기, 이끼진드기 등 다양한 절지동물들이 바이러스, 박테리아, 원충 등의 병원체를 숙주와 인간 사이에서 전파하는 매개체 역할을 합니다./ 예를 들어 말라리아, 뎅기열, 라임병 등 수많은 질병을 옮기는 매개체입니다./

둘째, 직접 병변을 일으키는 기생체로서의 중요성입니다. 일부 절지동물의 물림이나 분비물이 피부에 직접적인 손상과 병변을 일으킵니다./ 예를 들어, 심한 가려움과 염증을 동반하는 스가미귀의 가려움증, 모기의 물림 자국 등이 있습니다. 또한 해충에 물리면 2차 감염 가능성도 높아집니다./

따라서 이들 절지동물의 서식과 유행을 통제하는 것이 질병예방과 공중보건을 위해 매우 중요하다고 할 수 있습니다./

⇒ 전체적인 내용의 틀의 정확성과 관련성에는 문제가 없으나 이끼진드기, 스가미귀 등의 존재하지 않는 절지동물을 언급했다는 점에 있어서 정확성이 떨어지는 것으로 판단하였다.

<영어 - Claude Ai>

Volunteer arthropods play an important clinical role as vectors that transmit pathogens between hosts, resulting in human diseases./ Here is an explanation of their importance as disease vectors and causes of direct lesions:

1. As vectors:

- Many arthropods like mosquitoes, ticks, mites, etc. act as vectors and transmit pathogens like viruses, bacteria, protozoa between animal reservoirs and humans. /

- For example, mosquitoes transmit malaria, dengue and many other diseases. Ticks spread Lyme disease and mites spread scrub typhus between animals and people. /

- These arthropods pick up pathogens from infected hosts during a blood meal. The pathogens then multiply inside their bodies before getting transmitted to another host via bites or secretions. /

2. As causes of direct lesions:

- The bites or secretions released by certain arthropods directly result in skin sores and lesions. /

- For instance, scabies mites and some mosquito genera make skin burrows causing itchy mite/insect bite lesions. /

- Spider bites can necrotize tissue locally. Bee stings also elicit reactions causing pain and skin inflammation. /

- If the arthropods carry pathogenic organisms, these lesions may get secondarily infected worsening local symptoms. /

So in summary, arthropods like parasitic insects and arachnids play a huge clinical role - in transmitting dangerous illnesses to people as vectors and directly damaging skin tissue at bite/burrow sites. Their control is vital for public health. /

<한글 - Clova X>

임상적으로 중요한 의용절지동물은 벡터로서 인간에게 감염을 전파하는 역할을 합니다./ 의용절지동물은 다음과 같은 병원체를 매개할 수 있습니다.

- 리케치아: 발진티푸스, 쯔쯔가무시병, 렙토스피라증 등의 감염병을 매개합니다./
- 세균: 페스트, 콜레라, 매독 등의 감염병을 매개합니다./
- 바이러스: 황열, 뎅기열, 출혈열 등의 감염병을 매개합니다./

의용절지동물은 직접적인 병변을 일으키는 기생체로도 작용할 수 있습니다./ 대표적인 예로는 체체파리가 인간에게 수면병을 일으키는 경우가 있습니다./

의용절지동물은 인간의 건강에 매우 중요한 역할을 하며, 이들의 생태와 행동을 이해하고 관리하는 것이 중요합니다. 이를 통해 감염병의 예방과 치료에 큰 도움을 얻을 수 있습니다. /

⇒ 절지동물의 벡터로서의 역할을 전파시키는 병원체에 따라 분류하여 제시하였다. 그 중 매독을 매개한다고 나와있는데, 이는 사면발이가 매독을 매개한다고 잘못된 정보를 제시한 것으로 보인다. 사면발이는 매독의 vector가 아니며, 사면발이로 인한 가려움증으로 인해 피부를 긁어 상처가 생기면 그곳에 매독이 2차감염 되는 것으로 나타났기 때문에 이는 부정확한 정보였다.

또 기생체로서의 절지동물에 체체파리가 인간에게 수면병을 일으킨다고 나와있으나, “임상 기생충학(서울의대 저)”를 보면 체체파리가 수면병원층을 옮기는 것은 매개체로서의 절지동물이 발육증식형 전파를 하는 경우로, 기생체로 분류하여 소개한 것이 잘못되었다.

<영어 - Clova X>

말씀하신 내용에 대한 확실한 답변을 제공하기 어려운 상황입니다. 필요한 정보를 확인하여 조속히 답변드리도록 하겠습니다.

⇒ 영어로 질문하니 아예 답변이 불가하였다.

<한글 - Bing Ai>

의용절지동물은 인간의 건강과 생명을 위협하는 기생충이나 진드기 등을 말합니다./ 의용절지동물은 매개체로서와 직접 병변을 일으키는 기생체로서 모두 중요한 역할을 합니다./ 의용절지동물의 임상 중요성은 다음과 같습니다.

- 의용절지동물은 인간에게 다양한 질병을 전파할 수 있습니다. [예를 들어, 모기는 말라리아, 샤가스병, 수면병, 리슈만편모충증, 사상충증, 황열, 뎅기열 등의 질병을 매개하거나 원인체가 될 수 있습니다^1^](https://blog.naver.com/PostView.nhn?blogId=tsyong212&logNo=10089177842). 이러한 질병들은 인간의 면역력을 약화시시키고 합병증이나 사망에 이를 수 있습니다. /
- 의용절지동물은 인간의 생활환경과 밀접한 관련이 있습니다. [의용절지동물은 농업, 어업, 산업 등에서 인간의 먹이나 자원을 훼손하거나 오염시시킬 수 있습니다](https://blog.naver.com/PostView.nhn?blogId=tsyong212&logNo=10089177842)[^2^](http://www.endotoday.com/endotoday/arthropodology.html). 예를 들어, 벌집단은 식량생산에 영향을 주고, 거미집단은 가축이나 사람에게 위협을 줄 수 있습니다. /
- 의용절지동물은 인간의 문화와 역사에도 영향을 미칩니다. [의용절지동물은 인간의 신앙이나 예술에도 반영되기도 합니다](https://blog.naver.com/PostView.nhn?blogId=tsyong212&logNo=10089177842)[^3^](https://medicine.yonsei.ac.kr/medicine/research/researchcenter/tropics.do). 예를 들어, 기독교에서는 벌과 거미를 악마나 악당으로 비유하고, 공상과학에서는 기생충이나 진드기를 공포스러운 존재로 표현하기도 합니다. /

따라서 의용절지동물의 임상 중요성은 매우 큽니다. [의용절지동물의 분류, 생태/습성, 방제, 원인물질 규명 등에 관한 연구를 통해 절지동물 매개질병의 역학적 양상을 규명하고 예방 및 치료 방법을 개발함으로로써 국민보건 향상에 기여할 수 있습니다^2^](http://www.endotoday.com/endotoday/arthropodology.html)./

⇒파란색 글씨는 관련성과 정확도와는 무관한, bing에서 제공한 출처 하이퍼링크이다.

의용절지동물의 정의에 대해 물어본 것이 아닌데 그것을 답변한 것은 관련성이 떨어진다. 더불어 모든 의용절지동물이 인간의 건강과 생명을 위협하는 절지동물은 아님에도 불구하고 그러하게 표현한 것은 부정확한 정보이다. 해당 정보는 출처 확인 결과 네이버 블로그에서 제공된 정보인 것을 나타나 Bing ai의 신뢰도를 떨어뜨리는 근거가 되었다. 하지만 그 정보의 출처를 함께 제공하였으므로 이용자로 하여금 출처를 통해 정보의 정확성을 쉽게 의심하고 진위여부를 판단할 수 있다는 점에서 의의가 있다.

수면병과 리슈만편모충증은 모기가 아니라 파리로, 샤가스병은 kissing bug로 매개되는 질병이므로 부정확한 정보이다.

절지동물의 매개체와 기생체에 입각한 답변이 아니라 임상 중요성에 대하여 절지동물이 인간의 어떤 분야에 영향을 끼치는지를 중심으로 답변한 점이 아쉬웠다. 임상 중요성을 물어보았는데 인간의 생활환경과 식량생산에 끼치는 영향이나 인간과 문화의 역사에 끼친 영향을 답변한 것은 관련성이 떨어지는 답변으로 판단하였다.

<영어 - Bing Ai>

Arthropods are animals that have jointed legs and exoskeletons, such as insects, spiders, crustaceans, and centipedes. / Some arthropods can act as parasites, meaning they live on or inside another organism and cause harm. Other arthropods can act as vectors, meaning they carry and transmit diseases from one host to another. Arthropods can have both direct and indirect effects on human health. /

Direct effects of arthropods as parasites include:

- Causing skin lesions or infections by burrowing into the skin or injecting venom or saliva. [For example, scabies mites cause intense itching and rash; bed bugs cause allergic reactions and secondary bacterial infections; lice cause irritation and secondary bacterial infections^1^](https://academic.oup.com/labmed/article-abstract/25/7/429/2659202)[^2^](https://www.taylorfrancis.com/books/mono/10.1201/b22250/goddard-guide-arthropods-medical-importance-gail-miriam-moraru-jerome-goddard-ii). /
- Causing internal organ damage or bleeding by feeding on blood or tissues. [For example, ticks can transmit Lyme disease, Rocky Mountain spotted fever, babesiosis, anaplasmosis, ehrlichiosis, and other diseases; fleas can transmit plague; mosquitoes can transmit malaria, dengue fever, yellow fever, Zika virus, West Nile virus, and other diseases](https://academic.oup.com/labmed/article-abstract/25/7/429/2659202)[^3^](https://wwwnc.cdc.gov/eid/article/13/9/07-0706_article)[^4^](https://books.google.com/books/about/Physician_s_Guide_to_Arthropods_of_Medic.html?id=rWMeblT89DUC)./
- Causing allergic reactions or asthma attacks by triggering the immune system. [For example, some people may be allergic to bee stings or wasp stings; some people may be allergic to dust mites or cockroach droppings^5^](https://link.springer.com/referenceworkentry/10.1007/978-3-662-58713-3_23-1)./

Indirect effects of arthropods as vectors include:

- Transmitting infectious agents that can cause serious or fatal diseases in humans. [For example, mosquitoes can transmit dengue fever, yellow fever, Zika virus, chikungunya virus; ticks can transmit Lyme disease, Rocky Mountain spotted fever, babesiosis, anaplasmosis, ehrlichiosis; fleas can transmit plague; lice can transmit typhus](https://academic.oup.com/labmed/article-abstract/25/7/429/2659202)[^3^](https://wwwnc.cdc.gov/eid/article/13/9/07-0706_article)[^4^](https://books.google.com/books/about/Physician_s_Guide_to_Arthropods_of_Medic.html?id=rWMeblT89DUC)./
- Altering the behavior or ecology of the host species by competing for resources or influencing their reproduction. [For example, some insects may affect the pollination of plants; some spiders may affect the predation of other animals](https://academic.oup.com/labmed/article-abstract/25/7/429/2659202)[^6^](https://parasitesandvectors.biomedcentral.com/articles/10.1186/s13071-023-05887-x)./

Arthropods are important for the balance of nature and human well-being. However, they can also pose a threat to human health if they are invasive species that spread diseases to new areas or if they are overexploited for their products or services./ [Therefore, it is essential to monitor and control arthropod populations and diseases using various methods such as biological control agents (e.g., predators), chemical pesticides (e.g., insecticides), cultural practices (e.g., sanitation), personal protection (e.g., repellents), vaccination (e.g., anthrax), etc^7^](https://link.springer.com/chapter/10.1007/978-981-15-5483-4_20)/.

⇒파란색 글씨는 관련성과 정확도와는 무관한, bing에서 제공한 출처 하이퍼링크이다. 질문과 관련성이 떨어지는 절지동물의 정의에 대한 설명을 제공하였다.

기생체로서의 절지동물에서 두번째 설명의 예시는 매개체로서의 절지동물에 대해 설명하고 있어서 정확도가 떨어지는 정보로 판단하였다.

마지막에서 임상적 중요성을 나타내는 설명의 예시 중 백신에 탄저병에 대한 예시가 있는데, 탄저병은 절지동물과 관련이 없으므로 부정확한 정보로 판단하였다.

하지만 다른 질문을 했을 때와 마찬가지로 Bing은 제공하는 정보의 문장마다 출처 하이퍼링크를 제시하고 답변의 맨 아래에 출처 링크를 모아서 제시한다는 점에서 신뢰성과 그 정보의 정확성에 대해 쉽게 알아볼 수 있다는 장점이 있다.

<한글 - Bard>

의용절지동물은 인간의 건강에 다양한 영향을 미칠 수 있는 절지동물입니다./ 이 중에서 매개체로서의 역할과 직접 병변을 일으키는 기생체로서의 역할은 특히 중요한 임상적 중요성을 갖습니다./

의용절지동물의 매개체로서의 역할

의용절지동물은 병원균을 매개하여 질병을 전파할 수 있습니다. 예를 들어, 모기는 말라리아, 지카, 뎅기열 등의 질병을 매개하고, 진드기는 라임병, 에이즈, 중증열성혈소판감소증후군 등의 질병을 매개합니다./

의용절지동물의 매개체로서의 역할은 다음과 같은 측면에서 임상적으로 중요합니다.

- 질병의 전파: 의용절지동물은 다양한 질병을 전파하여 전 세계적으로 수백만 명의 사망자를 발생시키고 있습니다./
- 질병의 진단: 의용절지동물의 매개체로서의 역할을 이해하면 질병의 진단과 치료에 도움이 됩니다. 예를 들어, 모기에 물린 후 말라리아 증상이 나타나면 모기에 물린 후의 시간을 확인하여 말라리아를 진단할 수 있습니다./
- 질병의 예방: 의용절지동물의 매개체로서의 역할을 이해하면 질병의 예방에 도움이 됩니다. 예를 들어, 모기의 서식지를 제거하거나 모기 기피제를 사용하여 모기에 물리는 것을 예방할 수 있습니다./

의용절지동물의 직접 병변을 일으키는 기생체로서의 역할

의용절지동물은 직접적으로 인간에게 병변을 일으킬 수 있습니다./예를 들어, 벌은 독침을 사용하여 사람을 쏘아 독을 주입할 수 있고, 진드기는 피부에 기생하여 피부염이나 전신 질환을 일으킬 수 있습니다./

의용절지동물의 직접 병변을 일으키는 기생체로서의 역할은 다음과 같은 측면에서 임상적으로 중요합니다.

- 질병의 발생: 의용절지동물은 다양한 질병을 직접적으로 일으켜 인간의 건강을 위협하고 있습니다./
- 질병의 치료: 의용절지동물에 의해 발생한 질병은 적절한 치료를 통해 회복될 수 있습니다./
- 질병의 예방: 의용절지동물에 의해 발생한 질병을 예방하기 위해서는 의용절지동물에 대한 이해와 예방 조치가 필요합니다./

의용절지동물의 매개체로서와 직접 병변을 일으키는 기생체로서의 역할은 인간의 건강에 매우 중요한 영향을 미칩니다. 따라서 의용절지동물에 대한 연구와 이해를 통해 이러한 질병을 예방하고 치료하는 방법을 개발하는 것이 중요합니다./

⇒ 의용절지동물의 임상적 중요성에 대해서 기생체로서와 매개체로서 어떤 부분 때문에 중요한지를 체계적으로 분류하여 예시와 함께 제시하였다는 점이 인상깊었다.

의용절지동물의 매개체로서의 역할에서 진드기가 에이즈를 매개한다는 것은 부정확한 정보인 것으로 판단하였다.

<영어 - Bard>

Medical Arthropods: Carriers and Causers of Disease

Medical arthropods, like insects, spiders, and mites, play a crucial role in human health, impacting us in two primary ways:/

1. As Vectors of Disease:

They act as middlemen, transmitting pathogens like viruses, bacteria, and parasites from one host to another. This "vector-borne" transmission accounts for a significant portion of human infectious diseases globally./

Key points about arthropods as vectors:

- Diverse range of diseases: They spread diseases like malaria (mosquitoes), dengue fever (mosquitoes), Lyme disease (ticks), Chagas disease (kissing bugs), and West Nile virus (mosquitoes)./
- Significant public health impact: Vector-borne diseases cause millions of illnesses and deaths annually worldwide./
- Understanding transmission cycles: Studying the interaction between arthropod, pathogen, and human host is crucial for developing effective control strategies./

2. As Direct Causes of Lesions:

Some arthropods directly inflict harm on humans through bites, stings, or by burrowing into the skin. These lesions can range from mild irritation to severe allergic reactions and even systemic illnesses./

Key points about arthropods causing direct lesions:

- Bites and stings: Bees, wasps, scorpions, spiders, and some ants can inflict painful bites or stings, injecting venom that may cause localized pain, swelling, and allergic reactions./
- Skin infestations: Scabies mites burrow into the skin, causing intense itching and discomfort. Sandflies and bed bugs feed on human blood, leaving itchy welts./
- Allergic reactions: Dust mites, cockroaches, and some insect stings can trigger allergic reactions in sensitive individuals, ranging from mild hay fever to life-threatening anaphylaxis./

Clinical Importance:

Understanding the dual role of medical arthropods as vectors and direct lesion-causing agents is crucial for several reasons:/

- Disease prevention and control: By studying their biology and ecology, we can develop targeted interventions like insecticides, repellents, and habitat modification to control their populations and break the transmission cycle of vector-borne diseases./
- Diagnosis and treatment: Recognizing the characteristic lesions or symptoms caused by specific arthropods helps healthcare professionals diagnose and treat infections or allergic reactions promptly./
- Public health education: Raising awareness about the risks associated with arthropod bites, stings, and infestations empowers individuals to take preventive measures and seek medical attention when needed./

Conclusion:

Medical arthropods, though small, have a significant impact on human health. Recognizing their dual role as disease vectors and direct lesion-causing agents is crucial for developing effective prevention, control, and treatment strategies. By studying their biology and behavior, we can work towards minimizing the burden of arthropod-borne diseases and protecting ourselves from their harmful effects.

Remember, prevention is key! Taking measures like using insect repellents, wearing protective clothing, and maintaining good hygiene can significantly reduce your risk of encountering and being harmed by medical arthropods./

⇒기생체와 매개체로서의 의용절지동물에 대해 예시와 함께 설명하였고 임상적 중요성에 대하여도 적용되는 분야별로 나누어 설명하였다. 이에 대해서 한 문단의 종합적인 결론까지 제공하는 것이 인상적이었다. 많은 양의 정보를 체계적으로 정리하여 제시하였다는 점에서 훌륭한 답변이였다.

<한글 - Wrnt>

의용절지동물은 임상적으로 중요한 역할을 두 가지 측면에서 수행합니다.

첫째, 그들은 매개체로서 전염성 질병을 전파하는 역할을 합니다./ 많은 질병은 절지동물을 통해 인간이나 동물에게 전염될 수 있습니다. 예를 들어, 모기는 말라리아와 뎅기열 같은 질병을 전파할 수 있습니다. 모기가 감염된 피를 흡혈한 후 다른 호스트에게 쏘면, 질병의 원인체가 전달되고 감염이 전파될 수 있습니다. 이는 전염병의 확산과 전파에 큰 영향을 미칩니다./

둘째, 의용절지동물은 직접적인 병변을 일으키는 기생체로서의 역할도 합니다. 일부 기생체는 인간이나 동물의 조직에 침투하거나 기생하여 직접적인 병변을 유발할 수 있습니다./ 예를 들어, 사람의 피부에 기생하는 진드기는 피부염이나 가려움증을 일으킬 수 있습니다./ 이러한 기생체는 간접적으로 질병을 전파하는 경우도 있으며, 그들의 존재는 질병 예방과 통제에 대한 중요한 고려 사항입니다./

따라서, 의용절지동물은 전염병의 예방과 통제를 위한 중요한 대상으로 간주됩니다. 이들 동물들과의 상호작용을 통해 전염병의 확산을 방지하고, 기생체로부터 보호하기 위한 적절한 예방법과 치료법을 개발하는 것이 중요합니다./

<영어 - Wrnt>

의학적으로 중요한 절지동물은 매개체로서의 역할과 직접적인 병변을 일으키는 기생충으로서의 역할 두 가지 측면에서 설명될 수 있습니다.

먼저, 의학적으로 중요한 절지동물은 매개체로서 전염성 질병을 전파하는 역할을 합니다. 일부 절지동물은 병원체를 운반하고 다른 호스트 동물에게 전파할 수 있습니다. /예를 들어, 모기는 말라리아와 뎅기열 같은 질병을 전염시킬 수 있습니다. 모기가 감염된 피를 흡혈한 후 다른 사람에게 쏘면, 질병의 병원체가 전달되고 감염이 전파될 수 있습니다. 이는 전염병의 확산과 전파에 큰 영향을 미치며, 의학적 중요성을 가지게 됩니다./

둘째, 의학적으로 중요한 절지동물은 직접적인 병변을 일으키는 기생충으로서의 역할을 합니다. 일부 기생충은 인간이나 동물의 조직에 침투하거나 기생하여 직접적인 병변을 유발할 수 있습니다./ 예를 들어, 사람의 피부에 기생하는 진드기는 피부염이나 가려움증을 일으킬 수 있습니다. 또한, 기생충이 내부에 침투하여 조직을 파괴하거나 유해한 물질을 분비할 수도 있습니다./ 이러한 기생충은 질병의 원인이 되며, 의학적으로 중요한 적절한 예방법과 치료법의 개발이 필요합니다./

따라서, 의학적으로 중요한 절지동물은 전염병 예방과 통제, 그리고 직접적인 병변의 치료와 예방에 큰 역할을 합니다. 이러한 동물들과의 상호작용을 통해 전염병의 확산을 방지하고, 기생충에 의한 질병을 예방하고 치료하는 방법을 개발하는 것이 중요합니다./

⇒ 한글로 질문하였을 때와 거의 비슷한 내용의 답변을 내놓았다.

3. 먼지진드기와 알레르기의 관계를 설명할 수 있어야한다.

<한글 - GPT 4.0> 정확한 정보

먼지진드기는 일반적으로 집먼지와 관련이 있으며, 이들은 사람의 피부 각질, 꽃가루, 곰팡이 등을 먹고 삽니다. / 먼지진드기의 배설물과 사체는 알레르겐으로 작용하여 알레르기 반응을 일으킬 수 있습니다. / 특히, 먼지진드기에 의한 알레르기 반응은 아토피 피부염, 알레르기성 비염, 천식 등 다양한 형태로 나타날 수 있습니다.

먼지진드기 알레르기의 증상으로는 재채기, 코 막힘, 눈 가려움, 피부 발진 등이 있을 수 있으며, 이러한 증상은 집먼지 노출 시 더욱 심해질 수 있습니다. /먼지진드기를 줄이기 위한 방법으로는 정기적인 청소, 침구류와 커튼을 자주 세탁하는 것, 습도 조절, 공기 청정기 사용 등이 있습니다.

/ 먼지진드기 알레르기가 의심되면 의사의 진단을 받고 적절한 치료를 받는 것이 중요합니다. 이는 알레르기 피부 반응 검사나 혈액 검사를 통해 확인할 수 있습니다.

<영어 - GPT 4.0>

Dust mites are tiny creatures commonly found in household dust. / They thrive in warm, humid environments and feed on dead skin cells shed by humans. / Dust mites themselves are not allergens, but their **feces** and **body fragments** can trigger allergic reactions in sensitive individuals. / This is because these particles contain proteins that are recognized by the immune system of allergic individuals as a threat, leading to an allergic reaction./

The association between dust mites and allergies is significant, particularly in cases of respiratory or skin allergies such as:

- **Asthma**: Dust mite allergens can trigger asthma attacks in individuals who are allergic to them. Symptoms can include wheezing, shortness of breath, chest tightness, and coughing. /
- **Allergic Rhinitis**: Commonly known as hay fever, this condition can be exacerbated by dust mites. Symptoms include sneezing, runny or stuffy nose, itchy eyes, nose, or throat, and watery eyes. /
- **Atopic Dermatitis**: Also known as eczema, dust mite allergens can worsen this skin condition, leading to itchy, inflamed, and sometimes blistered or weeping skin. /

To manage dust mite allergies, it's important to reduce exposure to dust mites. This can be achieved through various measures such as using allergen-proof bed covers, washing bedding in hot water regularly, maintaining low indoor humidity, vacuuming with a HEPA filter, and removing dust-collecting items like heavy curtains and plush toys from bedrooms. /

If someone suspects a dust mite allergy, it's advisable to consult a healthcare provider. They may recommend allergy testing and can suggest appropriate treatments, which may include antihistamines, nasal corticosteroids, or allergen immunotherapy. /

<한글 - Claude Ai>

먼지진드기와 알레르기의 관계에 대해 더 자세히 설명드리겠습니다.

• 먼지진드기는 0.2-0.3mm 크기의 극미소 절지동물로, 주로 침대, 소파, 카펫 등 집 안에서 서식합니다. /

• 먼지진드기의 배설물과 시체조각은 강력한 알레르기 반응을 일으키는 항원을 포함하고 있습니다. /

주요 항원은 Der p 1, Der p 2 등입니다. /

• 먼지진드기 알레르기는 알레르기성 천식 발병의 가장 큰 위험 인자 중 하나입니다. /

• 먼지진드기 알레르기 증상으로는 코막힘, 재채기, 눈이 가려움, 천명 등이 흔합니다. /

• 먼지진드기 알레르기 진단은 피부 반응 검사, 혈액 검사로 확인할 수 있습니다. /

• 먼지진드기를 줄이기 위해서는 침구 일주일에 한 번 이상 세탁, 집 청소, 공기 청정기 사용 등이 도움됩니다. /

• 알레르기 증상 완화를 위해서는 항히스타민, 스테로이드 제제 등의 약물이 사용됩니다. /

⇒ 틀린 정보도 아니고 관련성이 아예 없는 정보도 아님. 범용적 알레르기증상 완화 약물을 알려준 거기 때문. 하지만 먼지진드기의 경우 SQ-HDM를 사용한다고 말하는 것이 더 관련있는 답변인 것으로 판단하여 관련없는 답변 0.5개인 것으로 결과에 반영함.

(대한민국 약학정보원 참고- [아카리작스12SQ집먼지진드기 설하정 Acarizax 12SQ House Dust Mite Sublingual Tab. 의약품 정보 (health.kr)](https://www.health.kr/searchDrug/result_drug.asp?drug_cd=2018010300004) )

<영어 - Claude Ai>

Here is a brief explanation of the association between dust mites and allergies:

- Dust mites are tiny bugs that live in house dust and feed on dead skin cells shed by people and pets. /
- Dust mites are a very common indoor allergen. Their feces and body parts can trigger allergic reactions in sensitive people. / About 10-20% of people are allergic to dust mites. /
- People with allergies such as asthma, eczema, and rhinitis are more prone to be allergic to dust mites. When dust mite allergens are inhaled, they can cause sneezing, runny nose, watery eyes, and other allergy symptoms. /
- The proteins found in dust mite feces and body parts are the actual allergenic substances. These tiny particles become airborne easily and enter human bodies. /
- Exposure to high levels of dust mites is a common trigger for allergy and asthma flares in sensitized individuals. / Controlling dust mites and reducing exposure helps improve allergy symptoms. /

In summary, dust mites are tiny organisms that provoke IgE-mediated allergies in people prone to allergic conditions when inhaled. / Reducing dust mites in home environments can help alleviate allergy symptoms for sensitized individuals.

<한글 - Clova X>

먼지진드기는 알레르기 질환을 유발하는 주요 원인 중 하나입니다.

먼지진드기는 주로 침구류, 카펫, 소파 등의 섬유 제품에 서식하며, / 사람의 각질이나 먼지를 먹고 삽니다. / 이 과정에서 먼지진드기의 배설물과 사체가 발생하는데, 이 물질들이 사람의 호흡기나 피부에 노출되면 알레르기 반응이 일어날 수 있습니다. /

알레르기 반응은 재채기, 콧물, 코막힘, 기침, 천식, 피부염 등 다양한 증상을 유발할 수 있으며, 심한 경우에는 호흡곤란이나 아나필락시스(급성 알레르기 쇼크) 같은 심각한 증상이 나타날 수도 있습니다./

따라서, 먼지진드기로 인한 알레르기를 예방하기 위해서는 다음과 같은 방법들이 있습니다.

- **먼지진드기가 서식할 수 있는 환경을 제거**합니다.
- 침구류나 카펫 등의 섬유 제품을 자주 세탁하고, 햇볕에 널어 건조합니다.
- 집안 청소를 자주 하여 먼지를 제거합니다.
- 실내 습도를 50% 이하로 유지합니다. /
- **먼지진드기가 서식할 수 있는 제품을 사용하지 않습니다.**
- 먼지진드기가 서식하기 쉬운 베개나 쿠션 등의 제품을 사용하지 않습니다.
- 가능하면 천연 소재의 제품을 사용하고, 합성 소재의 제품은 사용을 최소화합니다. /
- **면역력을 강화**합니다.
- 충분한 수면과 휴식을 취하고, 규칙적인 운동을 합니다.
- 영양 균형을 유지하고, 비타민과 미네랄을 충분히 섭취합니다. /

먼지진드기로 인한 알레르기 증상이 심한 경우에는 병원에서 치료를 받는 것이 좋습니다. 병원에서는 약물 치료나 면역 치료 등의 방법을 통해 알레르기 증상을 완화할 수 있습니다. /

⇒ 실내 습도를 50%이하가 아니라 40% 이하로 유지한다고 쓰는 것이 더 바람직함. 하지만 50% 이하에 40% 이하가 포함된다는 점에서 틀린 정보는 아니므로 틀린 정보 0.5개로 산정함. <https://www.thermofisher.com/allergy/kr/ko/allergen-fact-sheets.html?allergen=dust-mite>

<영어 - Clova X> -영문 답변을 한글로 자동 번역하여 제공

먼지진드기는 알레르기 질환을 유발하는 주요 원인 중 하나입니다. 주로 침구류, 카펫, 소파 등의 섬유 제품에 서식하며, / 사람의 각질이나 먼지를 먹고 삽니다. / 이 과정에서 먼지진드기의 배설물과 사체가 발생하는데, 이 물질들이 사람의 호흡기나 피부에 노출되면 알레르기 반응이 일어날 수 있습니다. /

알레르기 반응은 재채기, 콧물, 코막힘, 기침, 천식, 피부염 등 다양한 증상을 유발할 수 있으며, 심한 경우에는 호흡곤란이나 아나필락시스(급성 알레르기 쇼크) 같은 심각한 증상이 나타날 수도 있습니다. /

따라서, 먼지진드기로 인한 알레르기를 예방하기 위해서는 다음과 같은 방법들이 있습니다.

- 먼지진드기가 서식할 수 있는 환경을 제거합니다.
- 침구류나 카펫 등의 섬유 제품을 자주 세탁하고, 햇볕에 널어 건조합니다.
- 집안 청소를 자주 하여 먼지를 제거합니다.
- 실내 습도를 50% 이하로 유지합니다. /
- 먼지진드기가 서식할 수 있는 제품을 사용하지 않습니다.
- 먼지진드기가 서식하기 쉬운 베개나 쿠션 등의 제품을 사용하지 않습니다.
- 가능하면 천연 소재의 제품을 사용하고, 합성 소재의 제품은 사용을 최소화합니다. /
- 면역력을 강화합니다.
- 충분한 수면과 휴식을 취하고, 규칙적인 운동을 합니다.
- 영양 균형을 유지하고, 비타민과 미네랄을 충분히 섭취합니다. /

먼지진드기로 인한 알레르기 증상이 심한 경우에는 병원에서 치료를 받는 것이 좋습니다. 병원에서는 약물 치료나 면역 치료 등의 방법을 통해 알레르기 증상을 완화할 수 있습니다. /

⇒여기까지는 한글로 질문했을 때의 답변과 정확히 일치하고, 질문과 관련성이 떨어지는 미세먼지에 관련된 정보를 제공함.

미세먼지와 진드기 간의 연관성에 대한 새로운 이해가 나타나고 있습니다. 미세먼지는 대기 중에 떠다니는 입자로, 진드기의 이동 경로에 영향을 미칠 수 있습니다. 특히 미세먼지가 공기 중에 떠다니면서 진드기의 이동을 촉진하고 확산시킬 수 있습니다.

변화된 환경은 진드기의 번식과 생존에 영향을 미쳐 진드기의 증가를 유발할 수 있습니다. 미세먼지와 진드기의 건강 영향은 호흡기 질환과 진드기 알레르기의 상호 영향을 미칠 수 있습니다. 이와 같은 이유로 미세먼지와 진드기 간의 연관성을 이해하고, 이에 대한 대처 방안을 마련하는 것이 중요합니다.

⇒ 실내 미세먼지가 먼지 진드기에 영향을 미친다는 내용의 정보인데, 이 추가정보의 출처가 ai 답변에 첨부되어 있다. 주소는 다음과 같다. h[ttps://m.blog.naver.com/thzvnr4434/223292577468](https://m.blog.naver.com/thzvnr4434/223292577468) 하지만 이 출처는 네이버 블로그로, 정확성이 의심되어 먼지 진드기의 이동에 실내 미세먼지가 영향을 미친다는 내용을 medscape와 학술자료에서 탐색해 보았으나 관련정보가 없는 것을 통해 첫번째 정보를 부정확하고 관련성이 없는 정보로 판단하였다. 두번째 정보는 정확성에는 문제가 없으나 관련성이 떨어지는 정보로 판단하였다.

<한글 - Bing Ai>

알레르기는 우리 몸의 면역 체계가 특정 물질에 과민하게 반응하는 것을 말합니다. / 먼 지진드기는 작은 곤충으로, / 우리의 침대나 소파, 카펫 등에 서식하며, 피부의 각질이나 땀 등을 먹고 삽니다. / 먼지진드기 자체는 알레르기를 일으키지 않지만, 먼지진드기의 배설물이나 사체 조각이 공기 중에 떠다니면서 호흡기나 피부에 접촉하면 알레르기 반 응을 일으킬 수 있습니다. / 알레르기 반응은 다음과 같은 증상으로 나타날 수 있습니다.

• 피부 증상: 가려움, 두드러기, 습진, 아토 피성 피부염 등 /

• 호흡기 증상: 콧물, 재채기, 코막힘, 기 침, 천명, 호흡곤란, 천식 등 /

• 기타 증상: 눈 가려움, 눈물, 결막염, 두통, 피로, 불면증 등 /

⇒먼지 진드기는 거미강에 속하는 절지동물로 곤충류가 아니다.

<영어 - Bing Ai>

Dust mites are tiny creatures that live in many homes throughout the world. They feed on dead skin cells and other organic matter, and produce allergens that can trigger allergic reactions in some people. [/ Dust mite allergy is one of the most common causes of year-round allergies and allergic asthma^1^](https://aafa.org/allergies/types-of-allergies/insect-allergy/dust-mite-allergy/). /

Some of the **symptoms** of dust mite allergy include:

- Sneezing
- Runny nose
- Itchy eyes
- Watery eyes
- Asthma attacks
- Skin rashes /

Dust mite allergy can be diagnosed by a doctor who will ask about your medical history, perform a physical exam, and may order some tests to confirm the diagnosis. Some of the **tests** include:

- **Skin prick test**: A small amount of dust mite extract is placed on your skin and you are asked to scratch if you are allergic. /
- **Blood test**: A sample of your blood is taken and tested for antibodies to dust mites. /
- **Allergy patch test**: A small amount of dust mite extract is applied to your skin and monitored for any reactions. /

The **treatment for dust mite allergy** depends on the severity of your symptoms and how they affect your quality of life. Some of the treatment options include:

- **Avoidance**: The best way to prevent or reduce dust mite exposure is to avoid contact with them. This means keeping your home clean, using dust-proof covers for your mattress and pillows, washing your bedding regularly in hot water, vacuuming frequently with a HEPA filter, and using air filters or humidifiers in your home. /
- **Medication**: If you have mild to moderate symptoms, you may benefit from taking antihistamines, decongestants, nasal sprays, eye drops, or leukotriene modifiers to relieve your symptoms. If you have severe asthma attacks or anaphylaxis (a life-threatening allergic reaction), you may need an epinephrine auto-injector (EpiPen) and corticosteroids to treat them. /
- **Immunotherapy**: This is a long-term treatment that involves exposing you to gradually increasing doses of dust mite allergens under medical supervision. The goal is to desensitize your immune system to the allergens and reduce your sensitivity over time. /

⇒두번째에 하이퍼링크가 들어간 정보는 Bing에서 자체적으로 친 밑줄로, 본 논문의 관련성 평가와는 관련이 없다.

영어로 질문한 Bing은 제공된 정보의 출처 링크를 첨부한다는 점에서 장점이 있다. 제공된 출처 링크는 다음과 같다. [Dust Mite Allergy | AAFA.org](https://aafa.org/allergies/types-of-allergies/insect-allergy/dust-mite-allergy/) , [The Role of Dust Mites in Allergy | Clinical Reviews in Allergy & Immunology (springer.com)](https://link.springer.com/article/10.1007/s12016-018-8693-0) , [Dust Mites | American Lung Association](https://www.lung.org/clean-air/indoor-air/indoor-air-pollutants/dust-mites) 이 source들은 모두 공신력 있는 사이트라는 점에서 Bing ai가 정확도가 높은 ai라고 판단할 수 있었다.

또한 Claud ai에서 말했던 것 처럼 치료방침에 범용적인 알레르기 반응 완화제에 대한 정보만 제공하고 SQ-HDM을 언급하지 않았으므로 관련성이 없는 것은 아니나 정보가 부족하다고 판단하여 관련성 없는 정보 0.5개로 판단하였다.

<한글 - Bard> 영어로 질문하였을 때와 동일한 답변을 한글로 번역하여 제공

먼지 진드기와 알레르기의 연관성은 매우 강하며, 먼지 진드기는 연중 알레르기와 알레르기성 천식의 가장 흔한 유발 요인입니다. 먼지 진드기는 미세한 생물로, 따뜻하고 습한 환경에서 번성하며, 침구, 카펫, 푹신한 가구, 봉제 인형 등에 흔히 서식합니다.

먼지 진드기와 알레르기의 연관성은 다음과 같이 설명할 수 있습니다.

1. 노출: 우리는 매일 먼지 진드기 알레르겐, 주로 그들의 죽은 몸과 배설물을 흡입합니다.
2. 감작: 일부 개인의 경우 이러한 노출은 면역 체계가 알레르겐에 대한 항체(IgE)를 생성하도록 유발하여 감작을 유발합니다.
3. 알레르기 반응: 이후 알레르겐에 대한 노출은 비만세포가 히스타민 및 기타 염증성 화학 물질을 방출하도록 유발하여 알레르기 증상을 유발합니다.

먼지 진드기 알레르기의 일반적인 증상은 다음과 같습니다.

- 콧물 또는 코 막힘
- 재채기
- 가려운 눈, 눈물
- 기침
- 천명
- 피부 자극

먼지 진드기와 알레르기의 연관성은 다음과 같은 몇 가지 방법으로 나타납니다.

- 유병률: 먼지 진드기 알레르기는 전 세계 인구의 약 20%를 앓고 있으며, 전 세계적으로 가장 흔한 알레르기 중 하나입니다.
- 알레르겐의 강도: 먼지 진드기 알레르겐은 매우 강력하며 매우 낮은 농도에서도 알레르기 반응을 유발할 수 있습니다.
- 연중 증상: 계절성 알레르기를 유발하는 꽃가루와 달리 먼지 진드기 알레르기는 집 안에 먼지 진드기가 지속적으로 존재하기 때문에 연중 증상을 유발할 수 있습니다.

먼지 진드기를 완전히 박멸하는 것은 불가능하지만, 노출을 줄이고 증상을 관리하는 데 도움이 되는 몇 가지 전략이 있습니다.

- 침구 및 매트리스를 알레르겐 방지 커버로 덮습니다.
- 침구 및 린넨을 자주 뜨거운 물로 세탁하십시오.(130°F 이상)
- HEPA 필터 진공 청소기로 정기적으로 청소하십시오.
- 가정의 습도를 제습기로 낮추십시오.
- 알레르겐 면역 요법을 고려하십시오.

먼지 진드기 알레르기가 의심되는 경우 의료 전문가와 상담하여 진단 및 개인화된 치료 권장 사항을 받는 것이 중요합니다.

⇒ 영어로 질문하였을 때와 동일한 답변을 한글로 번역하여 제공한다. 허나 영어로 질문하였을 때 각 정보의 문장의 출처를 링크해주었던 것과 달리, 한글로 질문하였을 때는 링크해주지 않는다는 점에서 Bard를 한글버전보다 영어버전으로 사용하는 것이 더 효과적이라는 것을 보여주는 사례이다.

<영어 - Bard>

The association between dust mites and allergies is extremely strong, with dust mites being the most common trigger of year-round allergies and allergic asthma. / They are microscopic creatures that thrive in warm, humid environments, commonly found in bedding, carpets, upholstered furniture, and stuffed toys. /

Here's how the association works:

1. **Exposure**: We inhale dust mite allergens, primarily their dead bodies and droppings, on a daily basis. /
2. **Sensitization**: In some individuals, this exposure triggers the immune system to produce antibodies (IgE) against the allergens, leading to sensitization. /
3. **Allergic reaction**: Subsequent exposure to the allergens triggers mast cells to release histamine and other inflammatory chemicals, causing the classic allergy symptoms. /

Common symptoms of a dust mite allergy include:

- Runny or stuffy nose
- Sneezing
- Itchy, watery eyes
- Coughing
- Wheezing
- Skin irritation /

The strength of the association is evident in several ways:

- **Prevalence**: Dust mite allergy affects around 20% of the global population, making it one of the most common allergies worldwide. /
- **Allergen potency**: Dust mite allergens are highly potent and can trigger allergic reactions even at very low concentrations. /
- **Year-round symptoms**: Unlike seasonal allergies triggered by pollen, dust mite allergies can cause symptoms year-round due to their constant presence in our homes. /

While complete eradication of dust mites is impossible, several strategies can help reduce exposure and manage symptoms:

- Encase bedding and mattresses in allergen-proof covers.
- Wash bedding and linens frequently in hot water (at least 130°F).
- Vacuum regularly with a HEPA filter vacuum cleaner.
- Reduce humidity levels in the home using a dehumidifier. /
- Consider allergen immunotherapy to desensitize the immune system. /

If you suspect you have a dust mite allergy, it's important to consult a healthcare professional for diagnosis and personalized treatment recommendations.

⇒Bard ai의 특이점은 색깔로 제공된 정보의 출처를 각각 링크를 달아 제공하고 있다는 점이다.
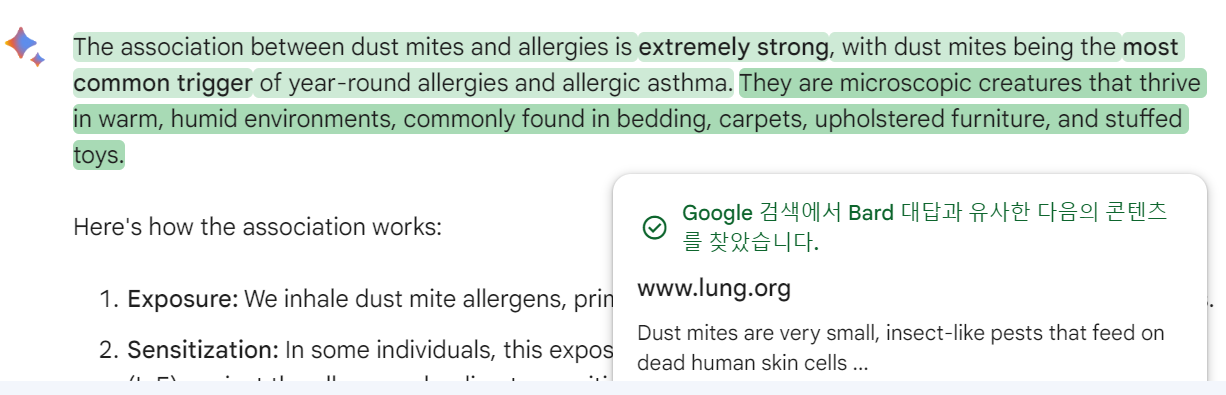


심지어는 틀린 google 검색에서 bard의 답변과 다른 정보가 있을 때 해당 google 검색 결과의 링크를 제시하여 이용자로 하여금 정보의 정확성을 비교하고 판단할 수 있도록 하는 기능이 있다는 장점이 있었다.


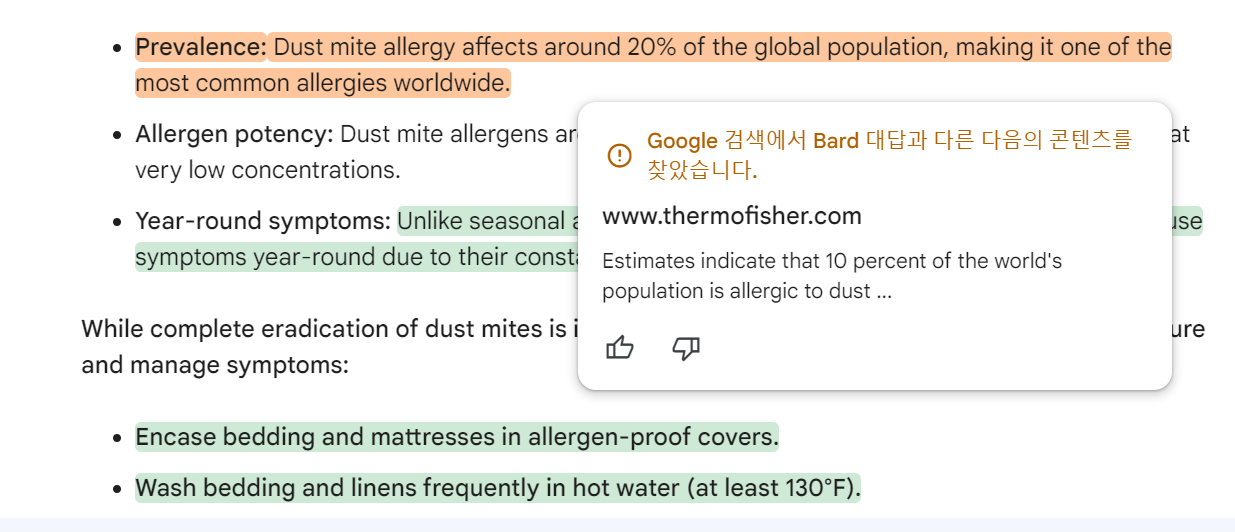


<한글 - Wrnt>

먼지진드기는 우리의 생활 환경, 특히 침구류, 카펫, 소파 등의 집먼지에 서식하는 아주 작은 곤충이에요. / 이 먼지진드기 자체는 인체에 직접적인 해를 끼치지 않지만, 그들이 분비하거나 폐사한 후 남은 물질이 알레르기 반응을 유발할 수 있어요. /

먼지진드기의 분비물과 사체에는 우리가 흔히 알레르기 증상으로 알고 있는 증상을 유발하는 단백질이 포함되어 있습니다. / 이러한 단백질들은 우리의 면역 시스템에 의해 외부 침입자로 인식되어, 이에 대한 반응으로 히스타민이라는 물질이 방출됩니다./ 히스타민의 방출은 알레르기 증상인 재채기, 코물, 눈물, 가려움증, 기관지의 수축 등을 유발합니다./

따라서, 먼지진드기 알레르기는 집에서 특히 많이 발생하며, 그 증상은 계절에 상관없이 일년 내내 나타날 수 있습니다. / 먼지진드기 알레르기를 예방하려면, 집안의 습도 조절, 정기적인 청소, 침구류의 세탁 등이 필요합니다. /

⇒Bing Ai에게 한글 질문했을 때와 같이 먼지 진드기를 곤충으로 표현하였는데, 거미강의 절지동물이라고 해야 한다.

<영어 - Wrnt> 한글로 답변을 자동 번역하여 제공함

먼지 진드기와 알레르기는 밀접한 연관성이 있습니다. 먼지 진드기는 가정 내에서 흔히 발견되는 작은 생물로, 우리의 가구, 카펫, 침구 등에서 살아갑니다. / 이들은 사람들의 피부와 머리카락, 집 안의 먼지에서 영양을 얻습니다. /

먼지 진드기는 알레르기를 유발하는 주요 원인 중 하나입니다. 먼지 진드기 알레르기는 호흡기 알레르기 증상을 일으킬 수 있는데, 이에는 코막힘, 콧물, 기침, 재채기, 눈 가려움, 눈물 등이 포함됩니다. / 또한, 먼지 진드기 알레르겐은 천식 환자들에게 더 큰 문제를 일으킬 수 있습니다. /

알레르기 증상을 줄이기 위해 먼지 진드기를 퇴치하려면, 정기적으로 침구를 세탁하고, 집을 청소하며, 공기 청정기를 사용하는 등의 방법을 시도해 볼 수 있습니다. /

4. 쯔쯔가무시병 매개 진드기의 학명을 댈 수 있어야 한다.

<한글 - GPT 4.0>

쯔쯔가무시병을 매개하는 진드기의 학명은 "Leptotrombidium deliense"입니다./ 이 진드기는 주로 동아시아와 남아시아 지역에서 발견되며, 쯔쯔가무시병을 일으키는 릭케치아(Rickettsia tsutsugamushi)를 사람에게 전파하는 역할을 합니다./

Leptotrombidium deliense는 쯔즈가무시를 전파할 수 있는 진드기의 일종임. 다만, 국내에서는 주로 발견되지 않으며 동남아에서 주로 발견되는 종임. 이를 통해 GPT가 한국 데이터 기반이 아님을 확인 할 수 있음. (<https://www.gidcc.or.kr/wp-content/uploads/2016/12/2016%EB%85%84-%EC%A7%84%EB%93%9C%EA%B8%B0%EB%A7%A4%EA%B0%9C-%EA%B0%90%EC%97%BC%EB%B3%91-%EA%B4%80%EB%A6%AC%EC%A7%80%EC%B9%A8.pdf>)

쯔즈가무시의 원인균은 리케치아종은 맞으나, 일반적으로 학명은 속-종으로 작성하므로 Orientia tsutsugamushi가 올바른 표기임.

<영어 - GPT 4.0>

The scientific name of the tick that serves as the vector for scrub typhus is "Leptotrombidium deliense"./ This tick is a significant carrier of the bacterium Rickettsia tsutsugamushi, which causes scrub typhus./

한국어 답변과 동일한 내용을 제공함.

<한글 - Claude Ai>

쯔쯔가무시병을 매개하는 진드기의 학명은 Ixodes persulcatus입니다./

Ixodes persulcatus는 Ixodes속 진드기의 일종으로, 시베리아, 몽골, 중국 동북부, 일본 홋카이도등 지역에서 발견됩니다. /

이 진드기가 리켓치아 입지아 균에 감염되면 사람이나 동물에 물려 쯔쯔가무시병을 옮길 수 있습니다./

따라서 Ixodes persulcatus 진드기는 쯔쯔가무시병의 주요 매개체로, 병의 예방과 통제를 위해 중요하게 다뤄지고 있습니다./

일반적으로 Ixodes persulcatus는 쯔쯔가무시보다는 라임병의 매개체로 알려져있음. (<https://synapse.koreamed.org/articles/1043190>)

<영어 - Claude Ai>

The scientific name of the tick vector that transmits Tsutsugamushi disease is Ixodes persulcatus./

In more detail:

- Scientific name: Ixodes persulcatus

- Common names: Taiga tick, Asian disease tick

- Genus: Ixode

- Family: Ixodidae/

Ixodes persulcatus is found in forests and wooded areas in Siberia, Mongolia, northeastern China, Japan's Hokkaido island, and other Far Eastern regions. /

This species of tick feeds on the blood of humans and other animals./ It can transmit the Rickettsia bacteria that causes Scrub typhus (Tsutsugamushi disease) to humans when infected during the blood meal./ Identifying the vector is important for disease prevention and control measures./

한국어 답변과 유사하게 주로 라임병을 전파하는 Ixodes persulcatus를 매개체로서 잘못 제시함.

<한글 - Clova X>

쯔쯔가무시병을 매개하는 진드기의 학명은 '오리엔티아 쯔쯔가무시(Orientia tsutsugamushi)'입니다. /

이 진드기는 알, 유충, 약충, 성충의 단계를 거치며, 주로 풀숲이나 밭 등의 야외에서 서식합니다. /

쯔쯔가무시병은 이 진드기의 유충이 사람을 물어서 감염되며, 발열, 두통, 오한 등의 증상이 나타납니다. /

가을철에 야외 활동을 할 때는 이 진드기에게 물리지 않도록 주의해야 합니다./

매개체의 학명이 아닌 원인균의 학명을 잘못제시함.

<영어 - Clova X>

The scientific name of the tick that transmits tsutsugamushi disease is 'Orientia tsutsugamushi'./

This tick goes through the stages of egg, larva, nymph, and adult, and is mainly found in grasslands and fields./

Tsutsugamushi disease is transmitted when the larvae of this tick bite humans, and symptoms include fever, headache, and chills./

When you are outdoors in the fall, you should be careful not to get bitten by this tick./

한국어 답변과 동일하게 매개체의 학명이 아닌 원인균의 학명을 잘못제시함.

<한글 - Bing Ai>

쯔쯔가무시병을 매개하는 진드기의 학명은 **Leptotrombidium akamusi**입니다[^1^](https://bing.com/search?q=%EC%AF%94%EC%AF%94%EA%B0%80%EB%AC%B4%EC%8B%9C%EB%B3%91+%EB%A7%A4%EA%B0%9C+%EC%A7%84%EB%93%9C%EA%B8%B0%EC%9D%98+%ED%95%99%EB%AA%85)./ 이 진드기는 쯔쯔가무시균에 감염되어 사람을 물면서 이 균을 전파합니다[^1^](https://bing.com/search?q=%EC%AF%94%EC%AF%94%EA%B0%80%EB%AC%B4%EC%8B%9C%EB%B3%91+%EB%A7%A4%EA%B0%9C+%EC%A7%84%EB%93%9C%EA%B8%B0%EC%9D%98+%ED%95%99%EB%AA%85). 이로 인해 쯔쯔가무시병이 발생하게 됩니다[^1^](https://bing.com/search?q=%EC%AF%94%EC%AF%94%EA%B0%80%EB%AC%B4%EC%8B%9C%EB%B3%91+%EB%A7%A4%EA%B0%9C+%EC%A7%84%EB%93%9C%EA%B8%B0%EC%9D%98+%ED%95%99%EB%AA%85)./ 이 진드기는 주로 야외 활동을 하는 사람들에게 물리는 경우가 많으며, 특히 논밭이 많은 지역에서 야외 활동을 하면서 흔히 물리게 됩니다[^1^](https://bing.com/search?q=%EC%AF%94%EC%AF%94%EA%B0%80%EB%AC%B4%EC%8B%9C%EB%B3%91+%EB%A7%A4%EA%B0%9C+%EC%A7%84%EB%93%9C%EA%B8%B0%EC%9D%98+%ED%95%99%EB%AA%85)/. 이 진드기에 물리지 않는 것이 쯔쯔가무시병을 예방하는 가장 좋은 방법입니다[^1^](https://bing.com/search?q=%EC%AF%94%EC%AF%94%EA%B0%80%EB%AC%B4%EC%8B%9C%EB%B3%91+%EB%A7%A4%EA%B0%9C+%EC%A7%84%EB%93%9C%EA%B8%B0%EC%9D%98+%ED%95%99%EB%AA%85)./

일반적으로 일본에서 관찰되는 쯔쯔가무시 매개 진드기로 알려져있음. (<https://www.gidcc.or.kr/wp-content/uploads/2016/12/2016%EB%85%84-%EC%A7%84%EB%93%9C%EA%B8%B0%EB%A7%A4%EA%B0%9C-%EA%B0%90%EC%97%BC%EB%B3%91-%EA%B4%80%EB%A6%AC%EC%A7%80%EC%B9%A8.pdf>)


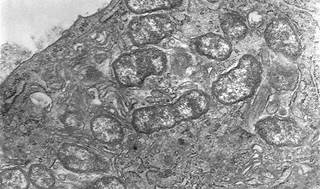


<영어 - Bing Ai>

The scientific name of the vector tick varies depending on the species./ For instance, the brown dog tick, which is found worldwide, is scientifically known as **Rhipicephalus sanguineus**^1^./ Another example is the deer tick, which is scientifically known as **Ixodes scapularis**[^2^](https://www.nationalgeographic.com/animals/invertebrates/facts/deer-tick)./ Please note that different species of ticks can transmit different diseases, so the vector tick for a specific disease may have a different scientific name./

Rhipicephalus sanguineus의 경우 일부 태국권에서 발견사례가 존재하긴하나,주로 발견되는 종은 아니며 Ixodes scapularis는 주로 라임병의 벡터로 알려져 있음.

전세계적인 분포보다는 태평양부근 국가에서 주로 발병함. ((<https://www.ncbi.nlm.nih.gov/pmc/articles/PMC5687757/>)

<한글 - Bard>

쯔쯔가무시병 매개 진드기의 학명은 Orientia tsutsugamushi입니다. /이 진드기는 털진드기과의 종으로, 사람과 동물에 기생하여 쯔쯔가무시병을 일으킵니다.

쯔쯔가무시병은 아시아, 오세아니아, 남아메리카 등지에 분포하는 풍토병으로, 매년 전 세계적으로 약 100만 건의 감염 사례가 보고되고 있습니다. 한국에서는 1940년대에 처음으로 보고된 이후, 매년 100~200건의 감염 사례가 보고되고 있습니다.

쯔쯔가무시병 매개 진드기는 주로 풀밭이나 숲속에서 서식하며, 사람과 동물을 물어 감염을 일으킵니다./ 진드기에 물린 후 약 1~3주 후에 발열, 두통, 근육통, 피부 발진 등의 증상이 나타납니다. 심한 경우 사망에 이를 수도 있습니다.

쯔쯔가무시병을 예방하기 위해서는 야외 활동7 시 진드기에 물리지 않도록 주의해야 합니다. 진드기에 물린 경우 즉시 병원을 방문하여 진료를 받아야 합니다.

쯔쯔가무시 매개체의 학명이 아닌 쯔쯔가무시 자체의 학명을 제시함.

국내 통계에 따르면 국내에서 쯔쯔가무시는 최근기준 매년 약 9000건 정도 보고되고 있음.

(<https://www.gidcc.or.kr/wp-content/uploads/2016/12/2016%EB%85%84-%EC%A7%84%EB%93%9C%EA%B8%B0%EB%A7%A4%EA%B0%9C-%EA%B0%90%EC%97%BC%EB%B3%91-%EA%B4%80%EB%A6%AC%EC%A7%80%EC%B9%A8.pdf>)

<영어 - Bard>

While Orientia tsutsugamushi is the bacteria responsible for causing tsutsugamushi disease, the scientific name of the vector tick that transmits it is Leptotrombidium deliense./ It's a species of chigger mite belonging to 3the Trombiculidae family./

Leptotrombidium deliense는 쯔즈가무시를 전파할 수 있는 진드기의 일종임. 다만, 국내에서는 주로 발견되지 않으며 동남아에서 주로 발견되는 종임. 이를 통해 Bard가 한국 데이터 기반이 아님을 확인 할 수 있음.

<한글 - Wrnt>

쯔쯔가무시병을 매개하는 진드기의 학명은 리츠체니아 삼두짐균(Leptotrombidium deliense)입니다./ 이 진드기는 쯔쯔가무시병의 주요 벡터로 알려져 있으며, 인간과 짐승에게서 쯔쯔가무시병을 전파할 수 있습니다. /쯔쯔가무시병은 매개체인 이 진드기에 의해 전염되므로, 이 진드기의 통제와 예방이 중요합니다./

Leptotrombidium deliense는 쯔즈가무시를 전파할 수 있는 진드기의 일종임. 다만, 국내에서는 주로 발견되지 않으며 동남아에서 주로 발견되는 종임. 이를 통해 GPT가 한국 데이터 기반이 아님을 확인 할 수 있음.

<영어 - Wrnt>

The scientific name of the tick vector depends on the specific species of tick being referred to./ There are several species of ticks that can act as vectors for various diseases./ For example, the scientific name for the black-legged tick, which is a vector for Lyme disease, is Ixodes scapularis./ The scientific name for the American dog tick, a vector for Rocky Mountain spotted fever, is Dermacentor variabilis./ It is important to note that different tick species can act as vectors for different diseases, and their scientific names may vary accordingly./

쯔쯔가무시 매개 벡터에 대한 질문이었는데, 쯔쯔가무시에 대한 정보는 없이 라임병, 록키산열 등의 정보만 제공하는 등 동문서답식 답변을 제시함.

5. 사람에 기생하는 이의 종류를 나열할 수 있어야 한다.

<한글 - GPT 4.0>

인간에 기생하는 이(lice)의 종류로는 크게 세 가지가 있습니다:

1. **머릿니 (Head lice)**: 머리카락에 서식하는 이들로, 주로 두피 부근에서 발견됩니다. 머릿니는 사람과 밀접한 접촉을 통해 전파되며, 특히 어린이들 사이에서 흔히 발생합니다.

2. **옷니 (Body lice)**: 이들은 옷과 인체 사이, 특히 몸통 부분에 서식합니다. 옷니는 불결한 환경과 밀접하게 관련이 있으며, 위생 상태가 나쁜 환경에서 더 흔하게 발견됩니다. 옷니는 때때로 질병을 전파하는 매개체가 될 수 있습니다.

3. **음모니 (Pubic lice)**: 이들은 주로 사람의 음모에 서식하지만, 때로는 다른 신체의 거친 모발, 예를 들어 겨드랑이, 가슴털, 심지어 눈썹과 속눈썹에서도 발견될 수 있습니다. 음모니는 성적 접촉을 통해 주로 전파되지만, 개인용품 공유를 통해서도 전파될 수 있습니다.

이러한 이들은 각각의 특징적인 서식지와 전파 방식을 가지고 있으며, 인간에게 불편함과 건강 문제를 일으킬 수 있습니다.

교과서상 머닛니는 머리의 두피에 기생하며, 옷니의 경우 옷에 서식하다가 흡혈시에만 몸으로 이동함.

<영어 - GPT 4.0>

Pediculus, commonly known as lice, are parasitic insects that can infest humans. There are three main types of lice that infest humans:

- Head Lice (Pediculus humanus capitis): These are the most common type and are found on the scalp. They are particularly common among children and are spread through direct head-to-head contact.
- Body Lice (Pediculus humanus corporis): These lice live in clothing and bedding and move to the skin to feed. They are larger than head lice and can spread diseases. Body lice infestations are often associated with poor hygiene and living conditions.
- Pubic Lice (Pthirus pubis): Also known as crab lice, they infest the hair and skin of the pubic area. They can also be found in armpits, eyelashes, and beards. Pubic lice are usually spread through sexual contact.

Each type of lice has a slightly different appearance and life cycle, but they all cause itching and discomfort. Treatment typically involves over-the-counter or prescription medications that kill lice and their eggs (nits). It's also important to wash clothing and bedding in hot water to prevent reinfestation.

<한글 - Claude Ai>

사람에 기생하는 주요 이의 종류는 다음과 같습니다:

- 검은머리이 - 머리에 기생하며 가려움을 유발할 수 있음
- 이지렁이 - 주로 머리가루에 서식하며 가려움을 유발할 수 있음
- 이상열성 이 - 피부에 굴을 파고 살면서 가려움과 염증을 유발할 수 있음
- 스케비스 - 주로 손톱과 발톱 사이에 서식함
- 선충 - 장에 기생하며 설사, 복통 등을 유발할 수 있음

이 외에도 여드름 이, 데모덱스 애카리 등 다양한 종류의 이가 사람의 피부, 모공, 머리카락 등에 기생하고 있습니다. 이들은 대부분 가려움, 염증 등을 유발하며 적절한 치료가 필요합니다.

정확한 의미를 알 수 없는 각종 이의 종류를 제시함. 옴과 선충을 비롯한 이와 관련없는 정보도 제시함.

<영어 - Claude Ai>

There are three main types of parasitic lice that can infest humans:

- ﻿﻿Head lice (Pediculus humanus capitis) - These tiny wingless insects infest hair and feed on blood from the scalp. They are very common, especially in children. Head lice spread through close personal contact and by sharing items like hats, combs, pillows etc.
- ﻿﻿Body lice (Pediculus humans humanus) - These lice live in clothing and bedding and come out to feed on blood from the skin. They can spread diseases like typhus, trench fever, and relapsing fever. Body lice are associated with poor hygiene and overcrowding.
- ﻿﻿Pubic lice (Pthirus pubis) - Also known as crabs, these lice infest the pubic hair and other coarse hair on the body. They are spread through sexual contact and sharing bedding or clothing. Pubic lice do not spread disease but can cause itching and discomfort.

Treatment involves medicated shampoos, creams or lotions containing ingredients like permethrin, pyrethrins, or malathion. Clothing, bedding and other items may also need treatment or hot washing to kill any lurking lice or nits. Maintaining good hygiene can help prevent infestations from spreading.

<한글 – Clova X Ai>

사람에 기생하는 이는 다음과 같은 종류가 있습니다.

머릿니: 사람의 머리카락에 기생하는 이로, 피를 빨아먹고 삽니다.

몸니: 사람의 몸에 기생하는 이로, 피를 빨아먹고 삽니다.

사면발이: 사람의 음모나 겨드랑이 털에 기생하는 이로, 피를 빨아먹고 삽니다.

이외에도 사람에 기생하는 이로는 털이, 쥐이 등이 있습니다.

이는 사람의 건강에 해를 끼칠 수 있으며, 전염성이 강하기 때문에 주의해야 합니다. 이를 예방하기 위해서는 청결한 환경을 유지하고, 옷이나 침구류를 자주 세탁하는 것이 좋습니다. 또한, 머리를 자주 감고, 몸을 자주 씻는 것도 중요합니다.

교과서상에서 머릿니는 두피에 기생하며 몸니는 옷에 기생하다가 흡혈시에만 몸으로 이동한다고 되어있어 틀린 정보를 제시함.

<영어 – Clova X Ai>

사람에게 감염을 일으키는 이는 몸니(Pediculus humanus humanus), 머릿니(Pediculus humanus capitis), 그리고 사면발이(Phthirus pubis)가 있습니다.

몸니는 사람의 몸에 기생하는 이로, 옷의 섬유에 알을 낳고 숙주의 피를 빨아먹으며 살아갑니다. 머릿니는 사람의 머리카락에 기생하는 이로, 두피의 피를 빨아먹으며 살아갑니다.

사면발이는 사람의 음모에 기생하는 이로, 주로 성적 접촉을 통해 전파됩니다. 이의 한 종류인 프티루스 푸비스가 사면발이의 원인입니다.

이들은 가려움증을 유발하며, 심한 경우 이차적 세균 감염을 일으킬 수 있습니다. 따라서, 이가 발견되면 즉시 치료를 받는 것이 중요합니다.

영어로 질문하였으나 한국어로 응답함.

<한글 - Bing Ai>

1. 머릿니 (head louse; Pediculus humanus var. capitis): 농촌지역의 초등학생에서 상당히 높은 유행이 있습니다.
2. 몸니, 옷이 (body louse; P. humanus var. corporis): 많이 감소하였습니다.
3. 사면발이 (pubic louse, crab louse; Phthirus pubis)

이들은 모두 외부기생충으로 분류됩니다. 외부기생충은 숙주의 표면에 붙어 사는 기생충을 말합니다. 반대로 장내 기생충을 포함한 모든 숙주 내부에 사는 것들을 내부기생충이라고 합니다. 내부기생충 내에서도 두 가지 생존 방식이 있습니다. 세포간 기생 (intercellular) 과 세포내 기생 (intracellular)이 그 두 가지 생존 방식입니다.

<영어 - Bing Ai>

Some types of parasitic pediculus in humans are:

• Head louse (Pediculus humanus capitis): A wingless insect that lives on the human scalp and feeds on blood. It can cause itching, scratching, dermatoses, and allergies. It can also transmit the pathogens Rickettsia prowazekii (epidemic typhus) and Bartonella quintana (five-day fever) 1

• Body louse (Pediculus humanus humanus): A wingless insect that lives on the human clothing and fomites and migrates to the human body to feed on blood. It can cause itching, scratching, dermatoses, and allergies. It can also transmit the pathogens Rickettsia prowazekii (epidemic typhus).

Bartonella quintana (five-day fever), and Borrelia recurrentis (louse-borne relapsing fever).

• Pubic louse (Pthirus pubis): A wingless insect that lives on the human pubic hair and other coarse hair and feeds on blood.

It can cause itching, scratching, dermatoses, and allergies. It can also transmit the

pathogens Treponema pallidum (syphilis) and Haemophilus ducreyi (chancroid

Rickettsia prowazedii, Bartonella quintana는 머릿니가 아닌 몸니에 의해 전파된다고 알려졌임. 머릿니에 의한 전파 사례를 찾지 못함. 또한 사면발이의 경우 질병을 전파하는것으로 알려진 것이 없음. Treponema pallidum, Heamophilus ducreyi는 사면발이에 의해 전파되기 보다는 2차적인 피부손상에 의한 감염으로 인해 발생함. (<https://www.cdc.gov/parasites/lice/pubic/disease.html)>

<한글 - Bard>

사람에 기생하는 이의 종류는 크게 3가지로 나눌 수 있습니다.

머리이는 사람의 머리카락에 기생하는 이입니다. 크기는 약 2-3mm이며, 검은색 또는 갈색을 띠고 있습니다. 머리이는 피를 빨아먹기 때문에 가려움증, 두피염, 탈모 등의 증상을 유발할 수 있습니다.

몸이는 사람의 몸에 기생하는 이입니다. 크기는 약 1-2mm이며, 흰색 또는 회색을 띠고 있습니다. 몸이는 피를 빨아먹기 때문에 가려움증, 피부염 등의 증상을 유발할 수 있습니다.

음부이는 사람의 음부에 기생하는 이입니다. 크기는 약 0.5-1mm이며, 흰색 또는 회색을 띠고 있습니다. 음부이는 성관계를 통해 전염되며, 가려움증, 발진, 질염 등의 증상을 유발할 수 있습니다.

이 외에도 등이, 가려움증이, 사마귀이 등 사람에게 기생하는 이들이 있습니다.

한국에서 가장 흔한 이의 종류는 머리이입니다. 머리이는 주로 비위생적인 환경에서 발생하며, 어린이에게 많이 발생합니다. 몸이는 머리이보다 덜 흔하지만, 전 세계적으로 널리 분포되어 있습니다. 음부이는 성관계를 통해 전염되기 때문에, 성생활이 활발한 성인에게 많이 발생합니다.

이의 감염을 예방하기 위해서는 다음과 같은 예방수칙을 준수하는 것이 중요합니다.

- 머리이: 머리를 자주 감고, 빗을 함께 사용하지 않도록 합니다.
- 몸이: 옷을 자주 갈아입고, 깨끗한 옷을 입습니다.
- 음부이: 성관계 시 콘돔을 사용합니다.

이의 감염이 의심되는 경우, 병원을 방문하여 진료를 받는 것이 좋습니다.

교과서상 제시된 크기, 색과 다른 정보를 제시함. 음부이의 경우 번역의 이상으로 일반적으로 사면발이라는 한국어 용어를 제시함.

<영어 - Bard>

There are two main types of parasitic pediculus in humans:

- ﻿﻿Pediculus humans capitis, or head lice, are small, wingless insects that live on the human scalp. They lay eggs, called nits, which are attached to the hair shaft. Nits hatch after about 7-10 days, and the nymphs (baby lice) mature into adults within 7-10 days. Head lice are spread through head-to-head contact, or by sharing hats, combs, or brushes.
- ﻿﻿Pediculus humans corporis, or body lice, are larger than head lice and live in clothing and bedding. They lay eggs in the seams of clothing and bedding, which hatch after about 6-9 days. The nymphs (baby lice) mature into adults within 10-12 days. Body lice are spread through close contact with an infested person, or by sharing clothing or bedding.

Both head lice and body lice can cause itching, redness, and inflammation of the skin. They can also transmit diseases, such as typhus and relapsing fever.

Here are some lesser-known types of parasitic pediculus:

- ﻿﻿Pediculus humans pubis, or pubic lice, are also known as crabs. They are smaller than head lice and body lice, and they live in the pubic hair and other coarse body hair. Pubic lice are spread through sexual contact, or by sharing clothing or bedding.
- ﻿﻿Pediculus simiae, or monkey lice, are similar to human lice, but they can infest monkeys and apes. They can also infest humans, but this is rare.
- ﻿﻿Pediculus schaeffi, or sea lion lice, are similar to human lice, but they infest sea lions. They can also infest humans, but this is very rare.

If you think you or someone you know may have parasitic pediculus. it is important to see a doctor or other healthcare professional. They can diagnose the infestation and recommend treatment.

Pediculus simiae라는 존재하지 않는 학명을 제시함.

Pediculus schaeffi는 바다사자가 아닌 원숭이에서 주로 기생하는 이의 종류로 알려져 있음

<한글 - Wrnt>

사람에 기생하는 이에는 여러 종류가 있습니다. 주요한 몇 가지를 나열해 드리겠습니다:

1. 청충 (Phthirus pubis) - 인간의 체모에 기생하는 작은 이로, 주로 성기 부근에서 발견됩니다.
2. 옷빈 (Pediculus humanus humanus) - 인간의 의류에 기생하며, 특히 옷과 피부 사이에서 발견됩니다.
3. 머리빈 (Pediculus humanus capitis) - 인간의 두피에 기생하는 이로, 머리카락 근처에서 발견됩니다.
4. 이등견 (Sarcoptes scabiei) - 인간의 피부에 기생하여 가려움증을 일으키는 작은 이로, 사람의 피부에 직접 파고들어 알을 낳고 번식합니다.

이 외에도 다양한 종류의 기생이들이 있지만, 위에 나열한 것들이 가장 흔하게 발견되는 기생 이 종류입니다. 기타 기생 이에 대해 더 궁금하시면 알려주세요!

한국어 용어의 제시가 잘못됨. 진드기의 일종인 옴을 이의 한종류로 제시함.

사면발이의 경우 인간의 체모중에서도 음모를 비롯한 coarse hair에서 자라므로 너무 포괄적인 정보를 제시함.

<영어 - Wrnt>

인간에게 기생하는 일부 피부진드류 종류는 다음과 같습니다:

1. ﻿﻿﻿두루미진드 (Head lice): 두루미진드는 인간의 머리카락에 기생하는 작은 진드입니다. 두루미진드는 피부에서 혈액을 섭취하여 생존합니다. 두루미진드 감염은 주로 직접적인 접촉을 통해 전파됩니다.
2. ﻿﻿﻿옷진드 (Body lice): 옷진드는 인간의 의류에 기생하는 진드입니다. 옷진드는 의류에 붙어있는 동안 피부로 이동 하여 혈액을 섭취합니다. 옷진드는 주로 개인 위생 상태가 나쁜 환경에서 발생하며, 옷이나 침대와 같은 물건을 공유하 는 것으로 전파될 수 있습니다.
3. ﻿﻿﻿곤충진드 (Pubic lice): 곤충진드는 인간의 음부에 기생하는 진드로, 종종 "꼬집기"라고도 불립니다. 곤충진드는 성적 접촉을 통해 전파되며, 또한 옷이나 침대와 같은 물건을 통해 전파될 수도 있습니다.

이러한 진드 종류는 적절한 치료 없이는 계속해서 인체에 기생하여 불편과 가려움증을 유발할 수 있습니다. 따라서 진드감염이 의심되는 경우 의료 전문가와 상담하여 적절한 치료 방법을 받는 것이 중요합니다.

주로 직역과 관련된 번역 이상 문제가 제일 심함. 영어로 질문하였음에도 한국어로 답변을 줌

6. 이의 구제법을 설명할 수 있어야한다.

<한글 - GPT 4.0>

이(lice)를 구제하는 방법은 이의 종류와 서식 위치에 따라 다를 수 있습니다. 여기 일반적인 구제 방법을 소개합니다:

1. 머릿니 (Head lice)

- **이 특수 샴푸 사용**: 약국에서 구입할 수 있는 이 전용 샴푸를 사용합니다. 이들은 페트리나(Pyrethrin) 또는 퍼메트린(Permethrin)과 같은 활성 성분을 포함하고 있으며, 사용 지침을 따라야 합니다.

- **빗질 치료**: 이 특수 빗(Nit comb)을 사용하여 죽은 이와 알을 빗어내야 합니다. 이 과정은 여러 번 반복해야 할 수 있습니다.

- **환경 정화**: 이들은 사람의 두피 외에 살지 않기 때문에, 침구류나 옷 등을 세탁하는 것이 좋습니다. 뜨거운 물(60°C 이상)에서 세탁하거나 건조기를 사용하는 것이 효과적입니다.

2. 옷니 (Body lice)

- **개인 위생 유지**: 깨끗한 옷을 자주 갈아입고, 목욕이나 샤워를 정기적으로 하는 것이 중요합니다.

- **옷과 침구류 세탁**: 옷과 침구류, 수건 등을 뜨거운 물에서 세탁하고, 가능한 경우 높은 온도로 건조시켜야 합니다.

- **환경 청소**: 이들이 살 수 있는 환경을 청소하고 정돈해야 합니다.

3. 음모니 (Pubic lice)

- **약물 치료**: 음모니를 제거하기 위해 퍼메트린(Permethrin)이나 말라티온(Malathion)과 같은 약물을 사용할 수 있습니다. 이 약물들은 일반적으로 크림이나 로션 형태로 제공됩니다.

- **빗질 치료**: 이 특수 빗을 사용하여 죽은 이와 알을 제거합니다.

- **성 파트너와의 상호 치료**: 음모니는 성적 접촉을 통해 전파되므로, 파트너도 동시에 치료를 받는 것이 중요합니다.

- **개인용품 분리 사용**: 개인용품을 다른 사람과 공유하지 않는 것이 중요합니다.

이 구제 방법들은 일반적인 지침에 불과하며, 개인의 상황에 따라 전문가의 조언을 구하는 것이 가장 좋습니다. 의사는 증상과 개인의 건강 상태를 고려하여 가장 적절한 치료법을 제공할 수 있습니다.

영어로 제공된 답변의 번역수준으로 모든 정보가 일치함. 번역상의 비약도 보이지 않음.

<영어 - GPT 4.0>

Certainly! Here's an explanation of the methods commonly used for lice relief:

1. **Head Lice Relief**

- **Medicated Shampoos**: Over-the-counter lice treatment shampoos containing permethrin or pyrethrin are often used. These shampoos kill the lice, and it's important to follow the instructions carefully.

- **Combing**: A fine-toothed comb (nit comb) is used to remove lice and their eggs (nits) from the hair. This process might need to be repeated over several days to ensure all lice and nits are removed.

- **Washing Belongings**: Wash clothing, bedding, and other fabric items in hot water and dry them on a high heat setting to kill any lice that may have transferred.

2. **Body Lice Relief**

- **Improved Hygiene**: Regular bathing and changing into clean clothing are crucial. Body lice live in clothing, so changing clothes regularly can help eliminate them.

- **Washing Clothes and Bedding**: Just like with head lice, washing items in hot water and using a high-heat dryer setting can effectively kill body lice.

- **Environmental Cleaning**: Cleaning the living area is also important as body lice can fall off and reside in bedding, towels, and clothing.

3. **Pubic Lice Relief**

- **Topical Insecticides**: Lotions or creams containing permethrin, malathion, or other insecticides are used to treat the affected area. It’s important to apply these treatments according to the instructions.

- **Combing**: Using a nit comb to remove lice and eggs can be effective, especially after applying a medicated treatment.

- **Treating Sexual Partners**: Since pubic lice can be sexually transmitted, it's important for sexual partners to be treated simultaneously to prevent re-infestation.

- **Avoid Sharing Personal Items**: Avoid sharing bedding, clothing, or towels to prevent the spread of lice.

General Tips

- **Follow Treatment Instructions**: Over-the-counter and prescription treatments come with specific instructions. It’s crucial to follow these to ensure the effectiveness of the treatment.

- **Repeat Treatment if Necessary**: Some treatments may require a second application after a week or so to kill any newly hatched lice.

- **Consult a Healthcare Provider**: If over-the-counter treatments are not effective, a healthcare provider can prescribe stronger medication.

Remember, while these are general guidelines, individual cases may vary, and consulting a healthcare professional is often the best course of action for appropriate treatment.

다른 AI와 달리 이의 구제법이라는 포괄적인 질문에도 불구하고 직접 3종류의 이로 나누어 각 이별 구제법을 나누어 설명한것이 특징적임.

일반적으로 빗질의 경우 주로 머릿니에 사용하나, 음모에도 서캐제거를 위해 이용되기도 한다. (<https://www.mountsinai.org/health-library/diseases-conditions/pubic-lice>)

<한글 - Claude Ai>

이의 구제법에는 다음과 같은 것들이 있습니다:

1. 적절한 살충제 사용

- 이약이나 이를 대상으로 한 샴푸, 로션 등을 사용하여 죽이거나 구충할 수 있음

1. 손톱, 머리카락 정기적 관리

- 손톱과 머리카락을 잘 관리하여 이가 생활할 환경을 제거

1. 청결 유지

- 정기적으로 샤워하고 청결을 유지하여 이가 피부에 붙잡히는 것을 방지

1. 침구류 세탁 및 일광 소독

- 이가 서식하기 좋은 침구를 자주 세탁하고 일광소독

1. 동거 가족들의 함께 구제

- 한 사람이 구제해도 다시 감염될 수 있으므로 동거인 모두 함께 구제 진행

이외에도 원인 별로 적절한 치료가 필요할 수 있습니다. 가려움이 심하면 의사의 진료를 받는 것이 좋습니다.

일반적으로 몸니, 머릿니, 사면발이 모두 손톱에서 서식하는 것으로 알려진 이는 없으며 더불어 이의 예방 및 치료를 위해 손톱관리를 하는 사례또한 찾을 수 없었음. 중간에 번역투로 느껴지는 어색한 문장이 존재함.

<영어 - Claude Ai>

Here are some common lice relief methods:

1. Use medicated shampoos, creams, or lotions that contain insecticides to kill lice. Products with pyrethrins or permethrin are often effective. Follow instructions carefully.

2. Use a fine-toothed lice comb to manually remove lice and nits after treatment with a medicated product. Comb through small sections of the hair at a time.

3. Wash clothing, bed linens, towels, combs, brushes, hats, etc in hot water and dry them at high heat to kill lice and nits. Items that cannot be washed can be sealed in a plastic bag for 2 weeks.

4. Vacuum carpets, furniture, mattresses well to pick up any stray hairs or nits.

5. Soak combs and brushes in rubbing alcohol or medicated shampoo for 1 hour before reuse.

6. Avoid head-to-head contact with anyone who has lice. Don't share personal items like combs, hats, helmets etc.

7. Conduct regular checks for 2-3 weeks after treatment to ensure lice have been eliminated. Retreat if live lice are spotted.

Seeing a doctor may be recommended for severe cases or if home treatment fails. Practice diligent hygiene and cleaning to prevent recurring infestations.

<한글 – Clova X Ai>

이의 구제법은 다음과 같습니다.

- 청결한 환경 유지: 이를 예방하기 위해서는 청결한 환경을 유지하는 것이 중요합니다. 옷이나 침구류를 자주 세탁하고, 머리를 자주 감고, 몸을 자주 씻는 것이 좋습니다.
- 살충제 사용: 살충제를 사용하여 이를 제거할 수 있습니다. 살충제는 머릿니나 몸니를 제거하는 데 효과적입니다.
- 참빗 사용: 참빗을 사용하여 머릿니를 제거할 수 있습니다. 참빗은 머릿니를 제거하는 데 효과적이며, 전통적인 방법으로 사용됩니다.
- 전문가의 도움: 이의 제거가 어려운 경우에는 전문가의 도움을 받는 것이 좋습니다. 전문가의 도움을 받으면 보다 효과적으로 이를 제거할 수 있습니다.
- 전염 방지: 이를 제거한 후에는 다른 사람에게 전염되지 않도록 주의해야 합니다. 옷이나 침구류를 다른 사람과 함께 사용하지 않고, 다른 사람과 접촉할 때는 마스크를 착용하는 것이 좋습니다.

영어로 질문했을때와 동일한 내용의 정보를 제공함. 다만 특이점은 다른 AI와 달리 한국어 기반으로 느껴지는 ‘참빗'이라는 용어의 사용임.

<영어 – Clova X Ai>

Lice relief methods are as follows.

1. Maintain a clean environment: To prevent lice, it is important to maintain a clean environment. Wash clothes and bedding frequently, wash your hair often, and wash your body frequently.

2. Use insecticides: You can use insecticides to get rid of lice. Insecticides are effective in removing head lice and body lice.

3. Use a chambit: You can use a chambit to remove head lice. A chambit is effective in removing head lice and is used as a traditional method.

4. Professional help: If it is difficult to remove lice, it is recommended to seek professional help. Professional help can help you remove lice more effectively.

5. Prevent transmission: After removing lice, you should take precautions to prevent transmission to others. Do not share clothes or bedding with others, and wear a mask when in contact with others.

Clova X가 한국형 Ai라는 점이 명명백백하게 드러난 부분으로, 외국의 경우 참빗을 이가 많은 (fine tooth comb)등으로 표현하는데, 클로바 X의 경우 참빗이라는 단어가 없자 그냥 한국식 이름인 chambit으로 표현함. 이를 통해서 클로바의 경우 한국정보기반의 정보를 제공함을 파악할 수 있었음. 일반적으로 이의경우 접촉을 통해 전파되므로 비말을 차단하는 마스크 착용과는 무관함.

<한글 - Bing Ai>

이의 구제법을 질문하였을때, 동음이의어인 법적인 정보를 제공함. 따라서 질문을 바꾸어 절지동물 이의 구제법을 질문하여 다시 답변을 얻음


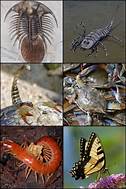


절지동물에게서 발생하는 문제를 해결하는 방법은 다양합니다. 절지동물이 인간의 생활 공간에 불편을 주는 경우, 그 해결책은 주로 방제에 초점을 맞춥니다[^1^](https://bing.com/search?q=%EC%A0%88%EC%A7%80%EB%8F%99%EB%AC%BC+%EC%9D%B4%EC%9D%98+%EA%B5%AC%EC%A0%9C%EB%B2%95).

1. 청결 유지: 실내를 청결한 상태로 유지하는 것이 중요합니다. 이는 절지동물이 우리의 생활 공간에 들어오는 것을 방지하는 첫 번째 단계입니다[^1^](https://bing.com/search?q=%EC%A0%88%EC%A7%80%EB%8F%99%EB%AC%BC+%EC%9D%B4%EC%9D%98+%EA%B5%AC%EC%A0%9C%EB%B2%95).
2. 입구 차단: 절지동물이 들어올 수 있는 곳의 입구를 막는 것도 중요합니다[^1^](https://bing.com/search?q=%EC%A0%88%EC%A7%80%EB%8F%99%EB%AC%BC+%EC%9D%B4%EC%9D%98+%EA%B5%AC%EC%A0%9C%EB%B2%95).
3. 살충제 사용: 절지동물이 돌아다닐 수 있는 통로에 겔이나 살충제를 사용하는 것이 효과적일 수 있습니다[^1^](https://bing.com/search?q=%EC%A0%88%EC%A7%80%EB%8F%99%EB%AC%BC+%EC%9D%B4%EC%9D%98+%EA%B5%AC%EC%A0%9C%EB%B2%95).

이러한 방법들은 절지동물로 인한 문제를 해결하는 데 도움이 될 수 있습니다. 그러나 이러한 방법들은 항상 절지동물의 생태와 생태계에 미치는 영향을 고려하여 신중하게 사용되어야 합니다. 절지동물은 자연 환경에서 중요한 역할을 하는 경우가 많으므로, 그들의 생태를 해치지 않는 방법으로 이의를 구제하는 것이 중요합니다[^2^](https://ko.wikipedia.org/wiki/%EC%A0%88%EC%A7%80%EB%8F%99%EB%AC%BC)[^3^](https://www.greelane.com/ko/%EA%B3%BC%ED%95%99-%EA%B8%B0%EC%88%A0-%EC%88%98%ED%95%99/%EB%8F%99%EB%AC%BC%EA%B3%BC-%EC%9E%90%EC%97%B0/marine-arthropod-facts-2291818/). 이러한 접근 방식은 절지동물로 인한 문제를 해결하는 동시에 생태계의 균형을 유지하는 데 도움이 됩니다[^2^](https://ko.wikipedia.org/wiki/%EC%A0%88%EC%A7%80%EB%8F%99%EB%AC%BC)[^3^](https://www.greelane.com/ko/%EA%B3%BC%ED%95%99-%EA%B8%B0%EC%88%A0-%EC%88%98%ED%95%99/%EB%8F%99%EB%AC%BC%EA%B3%BC-%EC%9E%90%EC%97%B0/marine-arthropod-facts-2291818/).

AI 답변에 이미지 정보도 함께 제시함.

이의 구제법을 법적인 용어로 인식하여 절지동물인 이의 구제법으로 질문하였는데, 질문의 포커스를 이가 아닌 절지동물에 맞추어 절지동물의 구제법에 대해 설명함. 또한 이러한 구제법은 이의 구제법과 거리가 있는 부정확한 정보임.

<영어 - Bing Ai>

Sure, here are some methods to relieve lice infestations:

1. **Confirm it is lice**: An itchy scalp may be caused by dandruff or dry skin rather than lice. Not all itchy, flaky scalps are due to lice. Before beginning a de-lousing regimen, a person should confirm that lice or their eggs are present^1^.
2. **Use a delousing shampoo**: There are a number of over-the-counter delousing shampoos available. Begin treatment with a delousing shampoo. Follow the instructions on the package, which may require the shampoo to remain on the scalp for several minutes^1^.
3. **Repeat treatment**: Some head lice shampoos kill both eggs and adult lice. These shampoos are called ovicides. More typically, shampoos have only a weak ovicidal effect or do not kill lice eggs at all. People who choose these shampoos will need to remove lice eggs by hand^1^.
4. **Remove nits with a comb**: After a shampoo treatment, the infected person should put on clean clothes. Machine washing clothes with hot water, or in a hot dryer, kills lice^1^.
5. **Clean sources of contagion**: Putting on dirty clothes can help the lice travel back to the head, reinfesting the scalp^1^.
6. **Home remedies**: Home remedies for lice may include olive oil, coconut oil, and mayonnaise. However, more scientific research is necessary to show whether these remedies are effective and safe^2^.

Please note that these methods are general suggestions and may not be suitable for everyone. Always consult with a healthcare provider for personalized advice^3^[^4^](https://www.healthline.com/health/lice/how-to-kill-head-lice)[^1^](https://www.medicalnewstoday.com/articles/318026).

1번 설명의경우 이의 구제법을 물었는데, 이임을 확인하라는 진단적인 관련없는 정보를 제시함.3과 4의 경우 표제로 제시한 내용과 구체적인 설명내용이 일치하지 않음. 6번내용의경우 의학적인 근거가 없는 민간요법에 가까운 내용을 제시함. 다만, 다른 AI와 달리 각 내용의 근거를 링크로 연결해 놓은것이 특징적임.

<한글 - Bard>

이의 구제법을 질문하였을때, 동음이의어인 법적인 정보를 제공함. 따라서 질문을 바꾸어 절지동물 이의 구제법을 질문하여 다시 답변을 얻었으나, 여전히 이와 관련된 법적인 정보를 제공함. 따라서 이의 치료및 예방법으로 다시 질문함. -> 한글 동음이의어에 대한 이해도가 낮으며, 이전에 5번 질문을 물어 자연스럽게 이해한 다른 AI와는 달리 이전 질문과의 연결력이 낮다고 느껴짐.

네, 알려드리겠습니다.

이의 치료

이의 치료는 원인에 따라 달라집니다.

- 감염성 이의 경우, 항생제를 투여합니다. 항생제는 세균이나 기생충의 감염을 치료하는 약물입니다.
- 알레르기성 이의 경우, 항히스타민제를 투여합니다. 항히스타민제는 알레르기 반응을 일으키는 히스타민의 작용을 막는 약물입니다.
- 자극성 이의 경우, 원인이 되는 자극을 제거해야 합니다. 예를 들어, 먼지 알레르기가 있는 경우, 집안을 청소하여 먼지를 제거해야 합니다.

이의 예방

이의 예방은 원인에 따라 달라집니다.

- 감염성 이의 경우, 손을 자주 씻고, 음식을 깨끗이 씻어 먹는 등의 개인 위생을 철저히 해야 합니다. 또한, 면역력을 높이는 것도 도움이 됩니다.
- 알레르기성 이의 경우, 알레르기 원인을 피하는 것이 가장 중요합니다. 또한, 항히스타민제나 비강 스프레이 등의 약물 치료를 통해 증상을 완화할 수 있습니다.
- 자극성 이의 경우, 원인이 되는 자극을 제거해야 합니다. 예를 들어, 먼지 알레르기가 있는 경우, 집안을 청소하여 먼지를 제거해야 합니다.

이의 치료 및 예방법에 대한 구체적인 내용은 다음과 같습니다.

감염성 이

감염성 이는 세균, 바이러스, 기생충에 의해 발생합니다. 세균성 이의 경우, 가장 흔한 원인은 포도상구균입니다. 바이러스성 이의 경우, 가장 흔한 원인은 헤르페스입니다. 기생충성 이의 경우, 가장 흔한 원인은 이구아나 이입니다.

감염성 이의 치료에는 항생제가 사용됩니다. 항생제는 세균의 성장을 억제하거나 죽이는 약물입니다. 바이러스성 이의 경우, 항바이러스제가 사용될 수 있습니다. 항바이러스제는 바이러스의 성장을 억제하는 약물입니다. 기생충성 이의 경우, 기생충을 죽이는 약물이 사용됩니다.

알레르기성 이

알레르기성 이는 꽃가루, 집먼지, 동물털, 음식물, 약물 등의 알레르기 원인에 의해 발생합니다. 알레르기 원인에 노출되면 면역 체계가 과민 반응을 일으키고, 이로 인해 이 증상이 나타납니다.

알레르기성 이의 치료에는 항히스타민제가 사용됩니다. 항히스타민제는 알레르기 반응을 일으키는 히스타민의 작용을 막는 약물입니다. 또한, 비강 스프레이나 인공눈물 등의 약물도 도움이 될 수 있습니다.

자극성 이

자극성 이는 먼지, 연기, 화학물질 등의 자극에 의해 발생합니다. 자극에 노출되면 눈의 점막이 손상되고, 이로 인해 이 증상이 나타납니다.

자극성 이의 예방에는 자극을 제거하는 것이 중요합니다. 예를 들어, 집안을 청소하여 먼지를 제거하고, 담배 연기를 피하고, 화학물질을 다룰 때는 마스크를 착용해야 합니다.

이의 치료 및 예방에 대한 자세한 내용은 의료 전문가와 상담하시기 바랍니다.

우선 이의 종류를 감염성이, 알레르기성이, 자극성 이와 같이 존재하지 않는 분류와 용어를 제시함. 일반적으로 우리가 알고 있는 이는 이중 그나마 전염이 된다는 점에서 관련된 정보를 감염성이로 제한해서 정보 판단을 진행함.감염성 이의 경우 퍼메트린과 같은 약물을 사용하는 것으로 알려져 있고 항생제를 1차적인 치료 약제로 사용하지 않음. 특히 기생충은 항생제로 사멸시킬 수 없음. 또한 이의 경우 접촉을 통한 전파가 이루어지는 것으로 음식물을 닦는것과는 예방방법이 멀음.더불어 이의 감염은 기생충인 이에 의해 발생한는것이지, 세균이나 바이러스, 이구아나가 원인이 아님.

<영어 - Bard>

I apologize for the confusion in my previous response. I understand you're looking for information on lice relief methods. Here are some effective approaches:

Over-the-counter (OTC) treatments:

- Permethrin (Nix): This lotion kills lice and their eggs (nits). Apply it to dry hair, leave it on for 10 minutes, and then rinse. Repeat the treatment in 7 days.
-
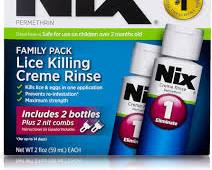

-
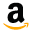
www.amazon.in
- Pyrethrin and piperonyl butoxide (Rid): This shampoo kills lice but not nits. Apply it to wet hair, lather, and leave it on for 10 minutes before rinsing. Repeat the treatment in 7 days.
-
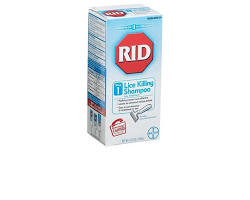

-
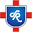
[www.saveritemedical.com](http://www.saveritemedical.com)
- Ivermectin (Sklice): This lotion kills lice and nits. Apply it to dry hair, leave it on for 10 minutes, and then rinse. One application is usually enough.
-
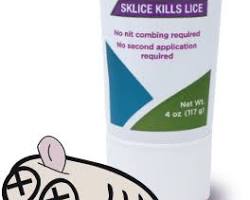

-
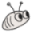
www.sklice.com

Physical methods:

- Wet combing: This method removes lice and nits physically. Use a fine-toothed nit comb on wet hair after applying conditioner. Comb from the roots to the ends of the hair, and wipe the comb frequently on a tissue. Repeat this process daily for 7-10 days.
-
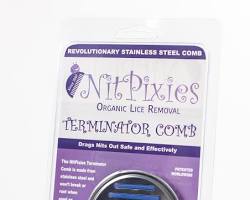

-
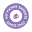
nitpixies.com
- Nit comb for lice removal

Home remedies (use with caution and consult a doctor before trying):

- Tea tree oil: This oil has insecticidal properties, but it can be irritating to the skin. Dilute it with carrier oil like coconut oil before applying it to the scalp.
-
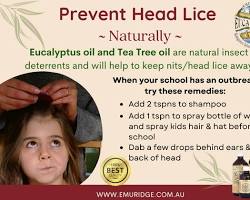

-
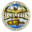
www.emuridge.com.au
- Mayonnaise: The thick consistency of mayonnaise can suffocate lice, but it's not as effective as other methods.

Additional tips:

- Wash all bedding, towels, and clothes in hot water (at least 130°F) and dry them on high heat to kill lice and nits.
- Vacuum carpets and furniture to remove any lice or nits.
- Avoid close head-to-head contact with others to prevent spreading lice.

Remember, it's important to consult a doctor if you have any concerns about lice or their treatment. They can recommend the best approach for your individual situation.

I hope this information is helpful!

RID의 경우 사용설명서 상에 젖은 머리가 아닌 dry hair에 사용하라고 표기되어 있음 (<https://medlineplus.gov/druginfo/meds/a601105.html>)

가정 치료방법으로 티트리 오일을 제시하였는데 일부 치료 성공사례 (<https://www.ncbi.nlm.nih.gov/pmc/articles/PMC3480584/>)가 보고 되고있긴하나, 여전히 더 연구가 필요한 부분으로 불확실한 정보라고 판단함. 더불어서 CDC의 경우 마요네즈와 오일과 같은 대증 요법은 과학적 근거가 부족하며 적절한 치료방법으로 권장하고 있지않으므로, 일반적인 정보 측면에서 정확하지 않은 정보라고 판단함. 일종의 민간요법에 가까움. [(https://www.cdc.gov/parasites/lice/head/gen_info/faqs_treat.html)](https://www.cdc.gov/parasites/lice/head/gen_info/faqs_treat.html))

또한 다른 AI보다 의학적인 약물치료 방법을 다수 제시하였으며 사진도 제공한다는 점에서 차별점이 나타남.

<한글 - Wrnt>

이들을 구제하는 방법은 종류에 따라 다르지만, 일반적인 구제법을 설명해 드리겠습니효곶다.

1. 청충 및 옷빈, 머리빈 구제법:
   - 이를 제거하기 위한 약물 치료: 이에 효과적인 약물 치료제가 있습니다. 흔히 사용되는 약물로는 페리트린, 말라티온 등이 있습니다. 처방 받아 사용하세요.
   - 빗질: 머리빈이나 옷빈을 제거하기 위해 특수한 이 빗을 사용하여 빗질하십시오. 이를 통해 이들을 물리적으로 제거할 수 있습니다.
   - 세탁: 기생 이가 있는 옷, 침구류 등은 뜨거운 물에 세탁하고, 건조기에서 높은 온도로 건조시키는 것이 좋습니다.
   - 진드기 방제 스프레이: 이가 있는 옷장, 침대, 가구 등에 진드기 방제 스프레이를 사용하여 기생 이를 제거할 수 있습니다.
2. 이등견 구제법:
   - 약물 치료: 이등견을 치료하기 위한 약물로는 페리트린, 이베르멕틴, 세더마린 등이 있습니다. 처방 받아 사용하세요.
   - 피부 관리: 감염된 부위를 깨끗이 닦고 가려움증이 줄어들 때까지 약물 치료를 계속해야 합니다. 가려움증이 심한 경우, 습진이나 피부염을 예방하기 위해 스테로이드 크림이 필요할 수도 있습니다.
   - 세탁: 기생 이가 있는 옷, 침구류 등은 뜨거운 물에 세탁하고, 건조기에서 높은 온도로 건조시키는 것이 좋습니다.

이 외에도 전문가의 도움을 받아 구제하는 것이 가장 좋습니다. 피부과 전문의와 상담하여 적절한 치료를 받으시길 바랍니다.

이의 종류에 대한 질문과 동일하게 한국어 기생충 용어사용이 부적절함. 또한 세더마린이라는 약물에 대한 정보는 존재하지 않음.

<영어 - Wrnt>

Here are some common lice relief methods, depending on the type of lice:

1. Treatment for pubic lice (crabs), body lice, and head lice:
   - Medication: There are effective treatments for lice, such as permethrin, malathion, and pyrethrin. Follow the instructions on over-the-counter products or get a prescription from a healthcare provider.
   - Combing: Use a fine-toothed lice comb to physically remove head lice or body lice. Comb through the hair, paying special attention to the areas behind the ears and the nape of the neck.
   - Washing: Launder clothing, bedding, and other items that may be infested with lice using hot water, followed by machine drying at a high temperature.
   - Insecticide sprays: Apply insecticide sprays to clothing, beds, and furniture to eliminate lice.
2. Treatment for scabies (itch mite):
   - Medication: Scabies can be treated with medications such as permethrin cream, ivermectin, and crotamiton. Follow your healthcare provider's instructions4 for proper usage.
   - Skin care: Keep the affected area clean and continue the medication until the itching subsides. If the itching is severe, a steroid cream may be needed to prevent secondary skin infections or eczema.
   - Washing: Launder clothing, bedding, and other items that may be infested with mites using hot water, followed by machine drying at a high temperature.

It's always best to seek professional help for lice treatment. Consult a dermatologist or healthcare provider for appropriate treatment options.

이의 구제법을 물었는데, 옴의 구제법을 추가적으로 제시하는 관련도가 낮은 정보를 제공함.

옴의 구제법중 가려운 증상이 계속될경우 스테로이드를 통해 이차 세균감염을 예방할 수 있다고 되어있는데, 일반적으로 스테로이드의 경우 염증, 면역 억제작용을 나타내 세균감염 예방이 아닌 가려움증 완화의 목적으로 사용되므로 잘못된 정보이다. 또한 스테로이드의 경우 crusted scarbies에서 쓰는 것이 일반적이며 이차 세균감염예방으로는 주로 항생제를 사용한다.

(<https://www.webmd.com/skin-problems-and-treatments/treatment-scabies>)

(<https://www.cdc.gov/parasites/scabies/treatment.html>)

7. 옴의 증상 진단, 치료법을 설명할 수 있어야한다.

<한글 - GPT 4.0>

옴(scabies)은 피부에 기생하는 옴 진드기(Sarcoptes scabiei)에 의해 발생하는 전염성 질환입니다. 옴 진드기는 피부 속으로 파고들어 번식하며, 이로 인해 가려움증과 발진이 발생합니다. 다음은 옴의 증상, 진단 방법, 그리고 치료법에 대한 설명입니다.

**증상**

- 심한 가려움증: 특히 밤에 심해지는 것이 특징입니다.

- 발진: 피부에 작은 물집이나 구진(작은 붉은 부위)이 생길 수 있습니다.

- 피부의 가는 선: 옴 진드기가 피부 속을 파고들면서 생기는 가는 회색 또는 흰색의 선이 보일 수 있습니다.

- 피부 궤양 또는 감염: 긁음으로 인해 발생할 수 있습니다.

**진단**

- 증상에 기반한 평가: 의사는 가려움증과 발진의 유형, 밤에 가려움증이 심해지는지 등을 평가합니다.

- 피부 검사: 발진이 있는 피부 부위를 검사하여 옴 진드기, 그들의 배설물, 또는 알을 찾습니다.

- 피부 긁기 검사: 영향을 받은 피부를 긁어서 채취한 시료를 현미경으로 검사하여 진드기의 존재를 확인합니다.

**치료**

- 의약품: 옴 치료에는 피부에 바르는 약물인 퍼메트린(Permethrin) 크림, 말라티온(Malathion) 로션, 또는 벤질 벤조에이트(Benzyl benzoate)가 사용됩니다.

- 구강 약물: 심한 경우, 이버멕틴(Ivermectin)과 같은 구강 약물이 처방될 수 있습니다.

- 가정 내 치료: 옴 진드기는 사람의 몸 밖에서도 생존할 수 있기 때문에, 환자의 옷, 침구류, 타월을 뜨거운 물에서 세탁하고 뜨거운 공기로 건조시키는 것이 중요합니다.

- 가족 및 밀접 접촉자 치료: 옴은 매우 전염성이 강하기 때문에, 환자와 밀접하게 접촉한 사람들도 동시에 치료를 받아야 합니다.

옴은 적절한 치료를 받으면 완치될 수 있는 질환입니다. 하지만 치료 후에도 몇 주 동안 가려움증이 지속될 수 있으며, 이는 정상적인 반응입니다. 만약 증상이 지속되거나 악화되면 의사의 조언을 구하는 것이 중요합니다.

옴진드기는 사람의 몸밖에서도 생존할 수 있다고 되어 있는데, 일반적으로 옴진드기는 몸밖에서는 2-3일 정도밖에 생존하지 못한다고 알려져 있다.

(대한피부과학회 <https://www.derma.or.kr/new/general/disease.php?uid=5170&mod=document>)

다만 본 답변에서는 2-3일간의 외부 생존을 통해 의류나 침구를 통한 전파가 가능하다라는 의미에서 제시한것이라고 생각되어 0.5점만 차감함.

<영어 - GPT 4.0>

Scabies is a contagious skin condition caused by the infestation of a tiny mite known as Sarcoptes scabiei. These mites burrow into the skin, causing a rash and intense itching. Here's a detailed explanation of the symptoms, diagnosis, and treatment of scabies:

**Symptoms**

- Intense Itching: This is often worse at night and can be severe.

- Rash: Small red bumps and blisters may appear, especially between the fingers, on the wrists, elbows, armpits, waist, and groin.

- Burrow Tracks: Thin, irregular burrow tracks made up of tiny blisters or bumps on your skin.

- Secondary Infection: Scratching can lead to secondary skin infections.

**Diagnosis**

- Clinical Examination: A doctor will examine the skin for signs of scabies, paying close attention to the characteristic rash and burrow tracks.

- Skin Scraping: A doctor may scrape a small section of affected skin and examine it under a microscope to look for mites, eggs, or mite feces.

- Symptom Assessment: The diagnosis is often made based on the presence of typical symptoms, such as intense itching that worsens at night.

**Treatment**

- Topical Scabicides: Medications such as permethrin cream (5%) or crotamiton are commonly prescribed. These are applied over the entire body from the neck down and left on for a prescribed period before washing off.

- Oral Medications: In more severe cases, or when topical treatments fail, oral ivermectin may be prescribed.

- Treating Close Contacts: It's essential to treat family members and close contacts, as scabies can spread easily, even if they show no symptoms.

- Environmental Measures: Washing bedding, clothing, and towels in hot water and drying them in a hot dryer can help eliminate mites from the environment. Non-washable items can be sealed in a plastic bag for several days to a week.

**Aftercare and Follow-Up**

- Itching may continue for some weeks after the treatment due to the allergic reaction to the mites, even though the mites are dead.

- A follow-up with the doctor might be necessary if symptoms persist or if new burrows or rash appear.

Prompt treatment of scabies is crucial to avoid complications and to prevent spreading it to others. It is important for individuals suspected of having scabies to consult a healthcare provider for accurate diagnosis and appropriate treatment.

한국어 답변과 유사한 내용이지만 보다 정밀한 내용을 제공함

<한글 - Claude Ai>

옴의 주요 증상, 진단 및 치료 방법은 다음과 같습니다.

증상

- 가려움증, 피부염

- 구진, 반점, 홍반 등의 피부병변

- 수면장애

진단

- 피부과 전문의 상담 및 피부 병변 확인

- 피부 채취나 혈액 검사로 옴 감염 확진

치료 방법

- 옴 약물 바르기 - 벤솔벤스 등의 약물 도포

- 구충제 복용 - 이버멕틴과 같은 구충제 복용

- 증상에 따른 추가 치료

- 항히스타민제, 스테로이드 크림 : 가려움증 완화

- 수면제 : 수면장애 호전

- 동거인 검사 및 치료하여 재감염 예방

정기적인 피부관리와 청결 유지가 예방에 도움됩니다.

증상이 심할 경우 피부과 의사의 조기 진료가 필요합니다.

증상으로 제시된 수면장애의 경우 옴진드기에 의한 1차적인 증상이라기 보다는 가려움으로 인한 2차적인 증상임(<https://www.nhsborders.scot.nhs.uk/media/197859/scabies-June-2014.pdf>)

진단방법으로서 혈액검사를 제시하였는데 일반적인 진단방법으로 보기 어려우며 혈액검사의 시도가 있었으나, 부적절하다라는 결과가 존재 (<https://www.ncbi.nlm.nih.gov/pmc/articles/PMC4619658/>) 병변근처의 피부검사가 보다 일반적인 진단검사방법임.

벤솔벤스라는 약품이 존재하지 않음. 일반적으로 옴의 치료법으로 알려진 BB로션은 benzyl benzoate (벤질 벤조에이트)이므로 잘못된 정보를 제시함.

<영어 - Claude Ai>

Here is an explanation of the symptoms, diagnosis, and treatment of scabies:

Symptoms

- Itchy rash and skin inflammation

- Burrows visible on the skin surface

- Pimple-like itchy blisters

- Intense itching, especially at night

- Sores from scratching

Diagnosis

- Physical exam by a doctor to look for signs of mites

- Skin scraping to identify mites under a microscope

- Skin biopsy in unclear cases

Treatment

- Permethrin cream or other scabicides applied to the skin

- Oral medications like ivermectin to kill mites

- Antihistamines and topical steroids to relieve itching

- All clothes, towels, bedding washed in hot water

- Family members should be treated at the same time to prevent recurrence

Prevention involves avoiding skin-to-skin contact with infected persons. Maintaining good personal hygiene and disinfecting living spaces may also help reduce reinfection risk. See a doctor if symptoms persist after treatment.

일반적으로 옴의경우 임상증상과 피부표면의 현미경 소견을 통해 진단하고 피부생검은 불가능한것은 아니나 임상에서 거의 사용하지 않음

(<https://dermnetnz.org/topics/scabies>)

<한글 – Clova X Ai>

옴은 개선충이라는 진드기가 피부에 기생하여 발생하는 질환으로, 심한 가려움증과 함께 피부 발진이 나타납니다.

다음과 같은 방법으로 옴을 진단하고 치료할 수 있습니다.

1. 진단

- 피부 발진과 가려움증이 있는지 확인합니다.

- 피부를 긁어서 피부 조직을 얻은 후 현미경을 통해 옴 진드기를 확인합니다.

2. 치료

- 항진균제 연고를 발라줍니다.

- 침구류를 소독합니다.

- 전염성이 강하므로 가족 구성원 모두 같이 치료해야 하며, 환자뿐만 아니라 접촉자 역시 2주 동안 격리조치 해야 합니다.

3. 예방

- 청결한 환경을 유지합니다.

- 옷이나 침구류를 자주 세탁합니다.

- 다른 사람과 접촉할 때는 마스크를 착용합니다.

옴은 전염성이 강하므로, 환자와 접촉한 사람은 반드시 동반 치료를 받아야 하며, 환자가 입었던 의류와 침구류 등도 반드시 삶아서 관리해야 합니다.

옴은 진균이 아닌 기생충에 해당하는 절지동물로 항진균제가 아닌 퍼메트린 등의 구충제를 도포해야하며, 옴 의심환자의 경우 치료제 도포 24시간 격리, 확진자의 경우 의사가 격리 해제 판단시까지 격리를 진행한다. 일반적으로 접촉자의 경우에는 예방적 도포제 치료제를 수행하고 2주간의 격리를 진행한다는 사실은 잘못된 정보임. (<https://www.derma.or.kr/new/EoS/doctor.pdf>)

또한 옴은 절지동물로서, 접촉에 의해 전파되고 호흡기를 통해 감염되지 않으므로 마스크 착용은 적절하지 않은 예방법임.

<영어 – Clova X Ai>

옴은 개선충이라는 진드기가 피부에 기생하여 발생하는 질환으로, 심한 가려움증과 함께 피부 발진이 나타납니다.

다음과 같은 방법으로 옴을 진단하고 치료할 수 있습니다.

1. 진단

- 피부 발진과 가려움증이 있는지 확인합니다.

- 피부를 긁어서 피부 조직을 얻은 후 현미경을 통해 옴 진드기를 확인합니다.

2. 치료

- 항진균제 연고를 발라줍니다.

- 침구류를 소독합니다.

- 전염성이 강하므로 가족 구성원 모두 같이 치료해야 하며, 환자뿐만 아니라 접촉자 역시 2주 동안 격리조치 해야 합니다.

3. 예방

- 청결한 환경을 유지합니다.

- 옷이나 침구류를 자주 세탁합니다.

- 다른 사람과 접촉할 때는 마스크를 착용합니다.

국내에서 사용되는 옴 치료 약제는 crotamiton 성분의 유락신, permethrin 크림이 있습니다. 약제를 바르는 방법이 조금씩 다를 수 있으며, 이상으로 옴 진드기 증상과 원인 치료법에 대하여 자세히 알아보았습니다.

우선, 영어로 질문을 제시하였음에도 한국어로 답변을 제공하였으며 특이점은 국내의 치료약제를 설명했다는 점으로 클로바X의 경우 국내 데이터베이스 기반임을 확인 할 수 있었음.

<한글 - Bing Ai>

옴의 증상, 진단 및 치료법에 대해 설명드리겠습니다:

1. **옴의 증상**: 옴의 가장 특징적인 증상은 심한 가려움증입니다. 이 가려움증은 특히 밤에 심해집니다^1^[^2^](https://doctornow.co.kr/content/magazine/cb0bc7f96ef148519cb5e323e0b9521d). 옴 진드기가 피부를 파고들면서 분비물을 내놓고, 이 분비물이 알레르기 반응을 일으켜 가려움증을 유발합니다^1^[^2^](https://doctornow.co.kr/content/magazine/cb0bc7f96ef148519cb5e323e0b9521d). 가려움증 외에도 작은 붉은 발진, 수포, 고름 등이 나타날 수 있습니다^1^[^2^](https://doctornow.co.kr/content/magazine/cb0bc7f96ef148519cb5e323e0b9521d).
2. **옴의 진단**: 옴은 특징적인 병변의 분포와 가려움증, 옴 진드기 굴의 발견, 가족력 등을 바탕으로 진단됩니다^1^[^2^](https://doctornow.co.kr/content/magazine/cb0bc7f96ef148519cb5e323e0b9521d)[^3^](https://blog.naver.com/PostView.nhn?blogId=hyouncho2&logNo=60196952151)[^4^](https://www.amc.seoul.kr/asan/mobile/healthinfo/disease/diseaseDetail.do?contentId=32469). 의사는 피부를 검사하고 필요한 경우 피부 표본을 채취하여 현미경으로 옴 진드기나 알을 관찰하여 확진할 수 있습니다[^2^](https://doctornow.co.kr/content/magazine/cb0bc7f96ef148519cb5e323e0b9521d)[^4^](https://www.amc.seoul.kr/asan/mobile/healthinfo/disease/diseaseDetail.do?contentId=32469).
3. **옴의 치료법**: 옴은 저절로 사라지지 않으므로 의사의 처방을 받아 약물 치료를 받아야 합니다[^5^](https://ko.wikihow.com/%EC%98%B4-%EC%B9%98%EB%A3%8C%ED%95%98%EB%8A%94-%EB%B2%95)[^6^](https://wellbeinghow.com/%EC%98%B4/). 퍼메트린 크림, 린데인 로션, 스피노사드 현탁액 등의 약물이 사용됩니다[^2^](https://doctornow.co.kr/content/magazine/cb0bc7f96ef148519cb5e323e0b9521d)[^6^](https://wellbeinghow.com/%EC%98%B4/). 이 약물들은 귀에서부터 시작해 턱을 따라 내려가면서 크림을 바르도록 합니다[^5^](https://ko.wikihow.com/%EC%98%B4-%EC%B9%98%EB%A3%8C%ED%95%98%EB%8A%94-%EB%B2%95). 치료가 잘 되었어도 가려움증은 몇 주간은 계속되기도 합니다[^2^](https://doctornow.co.kr/content/magazine/cb0bc7f96ef148519cb5e323e0b9521d).

이러한 정보는 일반적인 정보이며, 개인의 상황에 따라 달라질 수 있습니다. 따라서 옴에 대한 의심이 있거나 치료가 필요한 경우에는 반드시 의료 전문가와 상의하시기 바랍니다^1^[^2^](https://doctornow.co.kr/content/magazine/cb0bc7f96ef148519cb5e323e0b9521d).

<영어 - Bing Ai>

Sure, here’s an explanation of the symptoms, diagnosis, and treatment of scabies:

1. **Symptoms**: The hallmark symptoms of scabies include a rash and intense itching that gets worse at night^1^[^2^](https://www.mayoclinic.org/diseases-conditions/scabies/symptoms-causes/syc-20377378)[^3^](https://www.healthline.com/health/scabies). The rash itself can consist of tiny bites, hives, and bumps under the skin[^3^](https://www.healthline.com/health/scabies). Common sites for scabies include the wrist, elbow, armpit, nipple, penis, waist, buttocks, and area between the fingers[^3^](https://www.healthline.com/health/scabies).
2. **Diagnosis**: To diagnose scabies, a health care provider looks at the skin for symptoms of mites[^4^](https://www.mayoclinic.org/diseases-conditions/scabies/diagnosis-treatment/drc-20377383). The provider may also take a sample of the skin to look at under a microscope, which allows them to see if any mites or eggs are present[^4^](https://www.mayoclinic.org/diseases-conditions/scabies/diagnosis-treatment/drc-20377383)[^5^](https://www.cdc.gov/parasites/scabies/diagnosis.html)[^6^](https://cks.nice.org.uk/topics/scabies/diagnosis/diagnosis/).
3. **Treatment**: Scabies treatment involves killing the mites and eggs with a medicated cream or pill[^7^](https://www.cdc.gov/parasites/scabies/treatment.html)[^4^](https://www.mayoclinic.org/diseases-conditions/scabies/diagnosis-treatment/drc-20377383). Several creams and lotions are available by prescription[^4^](https://www.mayoclinic.org/diseases-conditions/scabies/diagnosis-treatment/drc-20377383). Your health care provider will likely ask you to apply the medication to your whole body, from the neck down, and leave it on for at least 8 to 14 hours[^4^](https://www.mayoclinic.org/diseases-conditions/scabies/diagnosis-treatment/drc-20377383). Sometimes, you may have to apply the lotion twice[^4^](https://www.mayoclinic.org/diseases-conditions/scabies/diagnosis-treatment/drc-20377383). More treatments may be needed if new symptoms appear[^4^](https://www.mayoclinic.org/diseases-conditions/scabies/diagnosis-treatment/drc-20377383).

Please note that these methods are general suggestions and may not be suitable for everyone. Always consult with a healthcare provider for personalized advice^1^[^2^](https://www.mayoclinic.org/diseases-conditions/scabies/symptoms-causes/syc-20377378)[^3^](https://www.healthline.com/health/scabies)[^7^](https://www.cdc.gov/parasites/scabies/treatment.html)[^4^](https://www.mayoclinic.org/diseases-conditions/scabies/diagnosis-treatment/drc-20377383).

<한글 - Bard>

옴의 증상

옴은 사람 피부에 기생하는 옴진드기에 의해 발생하는 피부 질환입니다. 옴진드기는 사람의 피부 표피층에서 구멍을 파고 들어가 사는 기생충으로, 암컷 옴진드기는 하루에 2~3개의 알을 낳습니다.

옴에 감염되면 다음과 같은 증상이 나타납니다.

- 가려움증: 옴의 가장 흔한 증상입니다. 특히 밤에 심해지는 경우가 많습니다.
- 피부 발진: 옴진드기가 구멍을 파고 들어간 부위에 붉은 반점이 나타납니다.
- 피부 궤양: 옴진드기의 구멍이 곪아서 궤양이 생기는 경우도 있습니다.

옴의 진단

옴의 진단은 다음과 같은 방법으로 이루어집니다.

- 임상 진단: 환자의 증상과 병변의 양상을 보고 진단합니다.
- 병리 검사: 긁어낸 피부 조직을 현미경으로 관찰하여 옴진드기의 알이나 유충을 확인합니다.

옴의 치료

옴의 치료는 옴진드기를 죽이는 약물로 이루어집니다.

- 국소 치료제: 퍼메트린, 린덴 등이 있습니다. 목에서 발끝까지 전신에 바르고 8~12시간 후 씻어냅니다.
- 경구 치료제: 이버멕틴이 있습니다. 일주일에 2회, 총 2회 복용합니다.

옴에 감염된 사람은 가족이나 동거인도 함께 치료해야 합니다. 또한, 환자의 침구, 옷, 수건 등을 뜨거운 물로 세탁하거나 고온 건조하여 옴진드기를 죽여야 합니다.

옴의 예방

옴의 예방은 다음과 같은 방법으로 이루어집니다.

- 개인 위생을 철저히 한다.
- 밀접한 접촉을 피한다.
- 감염된 사람의 침구, 옷, 수건 등을 접촉하지 않는다.

옴은 전염성이 강한 질환이므로, 위의 예방 수칙을 준수하여 감염을 예방하는 것이 중요합니다.

이버멕틴의 경우 옴에대해서 1회 경구투여 후 2주후에 반복 투여한다고 알려져 있음. (<https://www.drugs.com/dosage/ivermectin.html>)

<영어 - Bard>

Scabies: Symptoms, Diagnosis, and Treatment

Scabies is a contagious skin condition caused by tiny mites called Sarcoptes scabiei. These mites burrow into the skin, where they lay eggs and multiply. This triggers an allergic reaction in the human body, leading to the characteristic symptoms of scabies.

Symptoms:

- Intense itching: This is the most common and often most bothersome symptom of scabies. Itching tends to be worse at night, when the mites are more active.
- Tiny red bumps: These bumps appear around the burrows where the mites have dug in. They can be seen on the hands, wrists, elbows, underarms, buttocks, genitals, and between the fingers.
- Blisters: Small, fluid-filled blisters may form around the bumps or in areas of intense scratching.
- Crusting and scaling: In severe cases, the skin may become crusted and scaly, particularly in children and individuals with weakened immune systems.
- Scratches and scabs: Scratching due to the intense itching can lead to secondary infections and open sores.

Diagnosis:

- Medical history and physical examination: A doctor will typically ask about your symptoms, any recent contact with someone with scabies, and examine your skin for the characteristic bumps and burrows.
- Skin scraping: In some cases, a doctor may take a small sample of skin to examine under a microscope for the presence of mites or their eggs.

Treatment:

- Scabicides: These are topical medications that kill the mites and their eggs. Common scabicides include permethrin cream and crotamiton lotion. They are usually applied once or twice, with repeat applications after a week if necessary.
- Oral medications: In some cases, especially for severe or crusted scabies, a doctor may prescribe an oral medication such as ivermectin to kill the mites.
- Antihistamines: These medications can help relieve itching and prevent further scratching.
- Soothing lotions and moisturizers: These can help to soothe irritated skin and prevent dryness.

Important notes:

- All household members and close contacts of someone with scabies should be treated, even if they don't have symptoms, to prevent further spread.
- Bedding, clothes, and towels should be washed in hot water and dried on high heat to kill the mites.
- Itching may persist for several days after treatment, even if the mites are dead, due to the body's allergic reaction.
- Seek medical attention if you suspect you have scabies, as early diagnosis and treatment is important to prevent complications and further spread.

Remember, I am not a medical professional and this information should not be taken as medical advice. Always consult with your doctor for diagnosis and treatment of any medical condition.

가장 많은 양의 정보를 오류 없이 제공함.

<한글 - Wrnt>

물론입니다. 옴은 피부에 발생하는 감염성 질환으로, 주로 세균에 의해 유발됩니다. 옴의 주요 증상은 피부의 발적, 붉은 색상, 부어오름, 가려움 등이 있습니다. 또한 피부가 따가울 수도 있습니다.

옴의 진단은 주로 의사나 피부과 전문의에 의해 이루어집니다. 의사는 환자의 증상을 확인하고 필요한 경우 피부 거즈로 샘플을 채취하여 세균 분류 검사를 시행할 수도 있습니다.

옴의 치료법은 일반적으로 항생제 크림이나 연고를 피부에 바르는 것으로 시작됩니다. 항생제는 세균을 제거하고 염증을 완화시키는 역할을 합니다. 또한 적절한 피부 관리와 청결 유지도 중요합니다. 치료 중에는 감염이 퍼지지 않도록 감염 부위를 마찰이나 자극으로부터 보호해야 합니다.

하지만 중요한 점은 옴의 치료는 의사의 지시에 따라 이루어져야 하며, 자가치료는 삼가해야 한다는 점입니다. 정확한 진단과 적절한 치료를 위해 의료 전문가의 상담을 받는 것이 가장 좋습니다.

옴은 진드기에 의한 기생충질환인데, 세균질환이라는 잘못된 정보를 제공하고 있으며, 이후 진단과 치료에서도 세균의 진단과 치료에 초점을 맞춘 잘못된 정보를 제공함.

<영어 - Wrnt>

Certainly. Scabies is a contagious skin condition caused by tiny mites called Sarcoptes scabiei. These mites burrow into the skin and lay eggs, leading to intense itching and a characteristic rash. Scabies is usually transmitted through close skin-to-skin contact with an infected person.

The symptoms of scabies include severe itching, especially at night, and the appearance of small, red bumps or blisters on the skin. The rash commonly occurs in areas such as the wrists, elbows, armpits, genital area, and between the fingers.

To diagnose scabies, a doctor will typically examine the affected areas of the skin and look for signs of burrows or the presence of mites. In some cases, a skin scraping may be taken and examined under a microscope to confirm the diagnosis.

The treatment for scabies usually involves the use of prescription medication that kills the mites and their eggs. The most commonly prescribed medication is a topical cream or lotion containing permethrin or another scabicide. This medication is applied to the entire body from the neck down and left on for a specified period of time before being washed off.

In addition to medication, it is important to wash all clothing, bedding, and towels in hot water to kill any mites that may be present. Personal items that cannot be washed should be sealed in a plastic bag for several days to kill the mites. Close contacts and family members may also need to be treated, even if they do not have symptoms.

It is crucial to follow the treatment plan prescribed by a healthcare professional to ensure the complete eradication of the mites and prevent reinfestation. If you suspect you have scabies, it is recommended to seek medical advice for an accurate diagnosis and appropriate treatment.
